# Membrane Proteomics Reveals the Role of Plant-Derived Smoke Solution on Wheat Under Salt Stress

**DOI:** 10.3390/ijms27167344

**Published:** 2026-08-17

**Authors:** Setsuko Komatsu, Shafiq Ur Rehman, Hisateru Yamaguchi, Keisuke Hitachi, Kunihiro Tsuchida

**Affiliations:** 1 Faculty of Environment and Information Sciences, Fukui University of Technology, Fukui 910-8505, Japan; 2 Department of Biology, University of Haripur, Haripur 22620, Pakistan; 3 Department of Management Nutrition, Aichi Gakusen University, Okazaki 444-8520, Japan; hyamaguchi@gakusen.ac.jp; 4 Center for Medical Science, Fujita Health University, Toyoake 470-1192, Japantsuchida@fujita-hu.ac.jp (K.T.)

**Keywords:** membrane proteomics, plant-derived smoke solution, salt stress, wheat

## Abstract

Salt stress severely limits wheat growth and seed yield; however, the mechanisms underlying plant-derived smoke (PDS)-induced salt tolerance remain unclear. The present study performs membrane proteomics to clarify how PDS solution enhances salt tolerance in wheat. Immunoblot analysis of subcellular marker proteins confirms successful enrichment of membrane fractions. Principal component analysis shows that 200 mM NaCl markedly alters membrane-protein composition in wheat roots, whereas 2000 ppm PDS solution largely restores these changes even under salt stress. At the protein level, mitochondrial ascorbate peroxidase increases in roots under salt stress but decreases with PDS-solution treatment, while leaves show the opposite trend. Salt stress reduces ATP content and H^+^-ATPase abundance; PDS-solution treatment restores both to near-control levels. In contrast, aquaporin levels increase under salt stress but decline after PDS-solution application. In addition, the expression of *ammonium transporter* was downregulated significantly under salt stress but recovered with PDS-solution treatment. These results suggest that PDS solution may confer salt-stress tolerance to wheat by regulating energy metabolism, water permeability, and ammonium absorption in the root membrane.

## 1. Introduction

Global climate patterns are being adversely affected by global warming, and the resulting extreme weather events have a direct impact on agricultural productivity [1]. Plants encounter various stresses during the processes of growth and development, which can be specifically classified into biotic and abiotic stresses. Salinity is a major abiotic stress factor, which inhibits growth and production of wheat [2]. Salt stress occurs due to the excessive accumulation of soluble salts in the soil [2], which inhibits crop growth and seed yield by adversely affecting osmotic balance and ionic homeostasis [3]. Osmotic and ionic factors, which are induced by salt stress, are similar to other abiotic stressors that activate oxidative stress by elevating reactive oxygen species (ROS) [4,5]. While salt affects cell membranes through the overproduction of ROS, osmoregulators play an important role in protecting plants from oxidative stress caused by salt [6]. The main sign of excessive salt levels in the root zone of wheat is the occurrence of ion toxicities and imbalances [7]. Elevated salinity in the root of wheat leads the accumulation of toxic Na^+^ in the stems, promoting competitive absorption of Na^+^ relative to K^+^/Ca^2+^ at root absorption sites, ultimately decreasing the intercellular K^+^/Na^+^ and Ca^2+^/Na^+^ ratios [8]. This is consistent with the fact that salt-tolerant wheat restricts Na^+^ uptake while maintaining high intracellular K^+^/Na^+^ and Ca^2+^/Na^+^ ratios [9]. These reports indicate that salt stress affects wheat roots, especially their cell membranes.

Plants mitigate the toxic effects of salt through the activation of antioxidant enzymes, synthesis of antioxidant compounds, biosynthesis of osmoprotectants, ion homeostasis, and hormonal regulation under salinity [10]. Salt-mediated osmotic stress reduces the ability of plants to extract nutrients and water from the soil, leading to stunted growth [11]. Ionic toxicity induced by the high concentration of Na^+^/Cl^−^ increases membrane peroxidation, disrupts cellular homeostasis, and inhibits photosynthesis in plants [12]. In addition, salt stress induces the accumulation of ROS, resulting in oxidative damage and inhibition of plant growth [13]. Given these findings, modifying proteins located in the cell membrane is considered an effective approach to alleviating the burden caused by soil salinity and enhancing plant tolerance to salt stress.

Early responses to abiotic stresses at the molecular and cellular levels involve complex signaling cascades, including changes in membrane permeability, protein kinase-mediated phosphorylation, and transcription factor-regulated gene expression. Among these regulatory mechanisms, transcription factors play pivotal roles in plant adaptation to salt stress by controlling the expression of stress-responsive genes. In wheat and other crop species, several transcription factor families have been identified as key regulators of salt tolerance. The WRKY family is known to regulate plant growth, development, stress resistance, and various metabolic pathways. For example, *TaWRKY17* enhances salt tolerance by modulating genes associated with abscisic acid signaling, ROS scavenging, and stress responses, thereby improving antioxidant capacity [14]. Similarly, members of the NAC family are important regulators of salt-stress responses. Under salt-stress conditions, the *tanac26-d3.2* mutant exhibited reduced root length, volume, and biomass compared with the wild type, indicating that *TaNAC26-D3.2* is a promising candidate gene involved in salt tolerance [15]. The MYB transcription factor family has also been implicated in salinity tolerance. A novel MYB gene, *TtMYB1*, was shown to enhance salt tolerance in transgenic wheat through increased proline and soluble sugar accumulation as well as elevated antioxidant enzyme activity [16]. In addition, basic leucine zipper (bZIP) transcription factors play well-established roles in salt-stress regulation. Overexpression studies demonstrated that *TabZIP15* positively contributes to salt tolerance, suggesting that manipulation of its expression may improve plant performance under saline conditions [17]. Collectively, these findings highlight the critical roles of diverse transcription factor families in coordinating the molecular networks underlying salt-stress tolerance in wheat.

Plant-derived smoke (PDS) solution generated during fire promotes the seed germination, plant growth and stress tolerance of plant species in a variety of habitats [18,19]. Butanolides, such karrikins and cyanohydrin, are the main active compounds in PDS solution [20]. The PDS solution reduced salt stress by regulating energy metabolism, protein glycosylation, and cell wall construction, thereby improving soybean growth [21]. The effects of salinity stress on rice were ameliorated by the combined application of PDS solution and *Bacillus safensis*-primed seeds, in which seeds were pretreated with a bacterial suspension prior to germination. This treatment improved germination percentage, seedling growth, K^+^ and Ca^2+^ contents, and photosynthetic pigments, while reducing Na^+^ content, total soluble protein, proline, total soluble sugar levels, and ROS-scavenging enzyme activity [22]. Smoke solution obtained from burning vineyard pruning waste reduced cadmium toxicity in grapevine seedlings [23]. These reports suggest that the addition of PDS solution causes changes at the molecular level in plants, conferring stress tolerance.

The increased frequency and intensity of extreme weather events caused by global warming are leading to the problem of reduced wheat yields [24]. High salinity and other abiotic stressors significantly limit wheat productivity [25]. Wheat is one of the most essential food crops, a moderately salt-tolerant crop, and this salinity tolerance has been extensively studied [26]. PDS solution promoted the growth of wheat inhibited by salt stress through the regulation of energy metabolism and flavonoid metabolism [27]. Furthermore, PDS solution enhanced soybean root growth with the effect on membrane and nuclear proteins, particularly promoting transcription through RNA polymerase II expression and energy production through ATPase accumulation [28]. These reports indicated that PDS might be involved in salt-stress tolerance in membrane fractions of wheat root. In the present study, the molecular and biochemical mechanisms underlying salt tolerance in wheat induced by PDS solution were investigated, with a particular focus on membrane-associated proteins. A membrane proteomic approach based on liquid chromatography–tandem mass spectrometry (LC–MS/MS) was employed following the purification of membrane proteins. To validate the proteomic findings and further clarify the mechanisms underlying the positive effects of PDS on wheat growth under salt stress, protein accumulation was analyzed by immunoblotting, gene expression by quantitative real-time PCR (qRT-PCR), and metabolite changes were also examined. This study primarily provides a fundamental understanding of PDS-solution-mediated salt tolerance at the molecular level; additionally, the findings may contribute to the development of strategies to improve crop performance under saline conditions.

## 2. Results

### 2.1. Membrane Purification and Purity Check of Wheat Root

**Figure 1:**
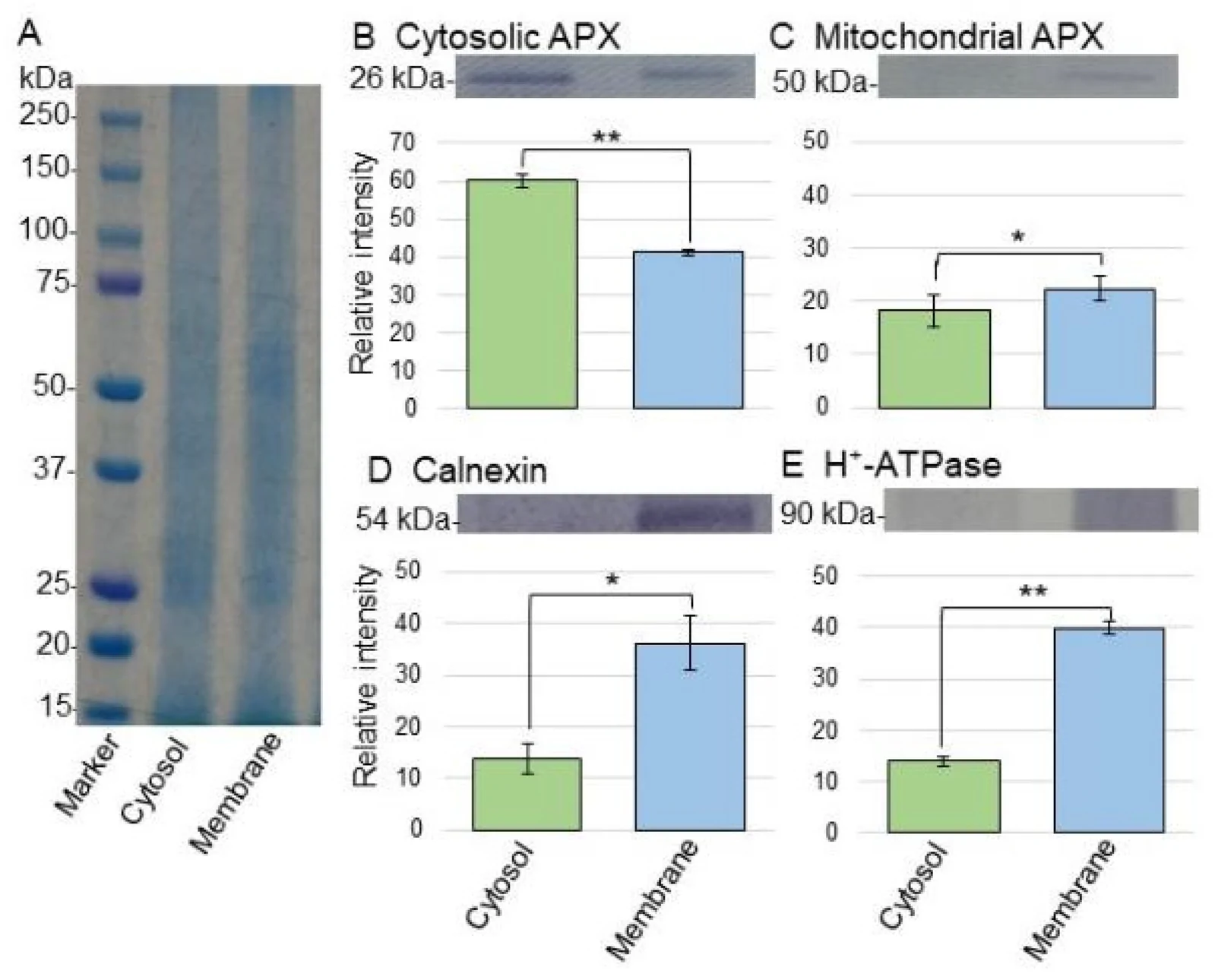
Purity evaluation of membrane fraction from wheat root. Proteins extracted from membrane fraction were evaluated using immunoblot analysis. The staining pattern of Coomassie brilliant blue was used as a loading control (**A**). In the immunoblot analysis, anti-cytosolic APX (**B**), mitochondrial APX (**C**), calnexin (**D**), and H^+^-ATPase (**E**) antibodies were used as protein markers of the cytosol, mitochondria, endoplasmic reticulum, and plasma membranes, respectively. Experiments were performed with 4 independent biological replicates (Figure S1). Data are shown as means ± SD from 4 biological replicates. Student’s *t*-test was used to compare values between control and treatment. Asterisk indicates a significant change (* *p* < 0.05, ** *p* < 0.01).

To investigate the subcellular function in wheat root treated with PDS solution, the membrane fraction was purified. Before analyzing the effects of PDS solution, a membrane-purification protocol was established. Roots of 5-day-old wheat were collected and the membrane fraction was purified. The purification efficiency of proteins extracted from the membrane fraction was evaluated by immunoblot analysis (Figure 1 and Figure S1). The staining pattern of Coomassie brilliant blue was used as a loading control (Figure 1A). Coomassie brilliant blue staining and protein concentration measurements by Bradford assay indicated that the protein concentrations were approximately equal in the cytosolic and membrane fractions (Figure 1A). In the immunoblot analysis, 26-kDa ascorbate peroxidases (APXs) (Figure 1B), 55-kDa APX (Figure 1C), 54-kDa calnexin (Figure 1D), and 90-kDa H^+^-ATPase (Figure 1E) were used as protein markers of cytosol, mitochondria membrane, endoplasmic reticulum membrane, and plasma membrane, respectively. H^+^-ATPase, 50-kDa APX, and calnexin were significantly accumulated in the membrane fraction compared with the cytosolic fraction (Figure 1C–E). On the other hand, the cytosolic fraction had higher accumulation of 26-kDa APX compared with the membrane fraction (Figure 1B). Based on the immunoblot analysis of subcellular-specific proteins, the membrane fraction was confirmed to be highly enriched for membrane proteins and was not contaminated with proteins from the cytosol. This purification protocol for the membrane fraction was used to investigate the subcellular function in wheat root treated with PDS solution under salt stress.

### 2.2. Identification and Functional Investigation of Membrane Proteins in Wheat Treated with PDS Solution Under Salt Stress

**Figure 2:**
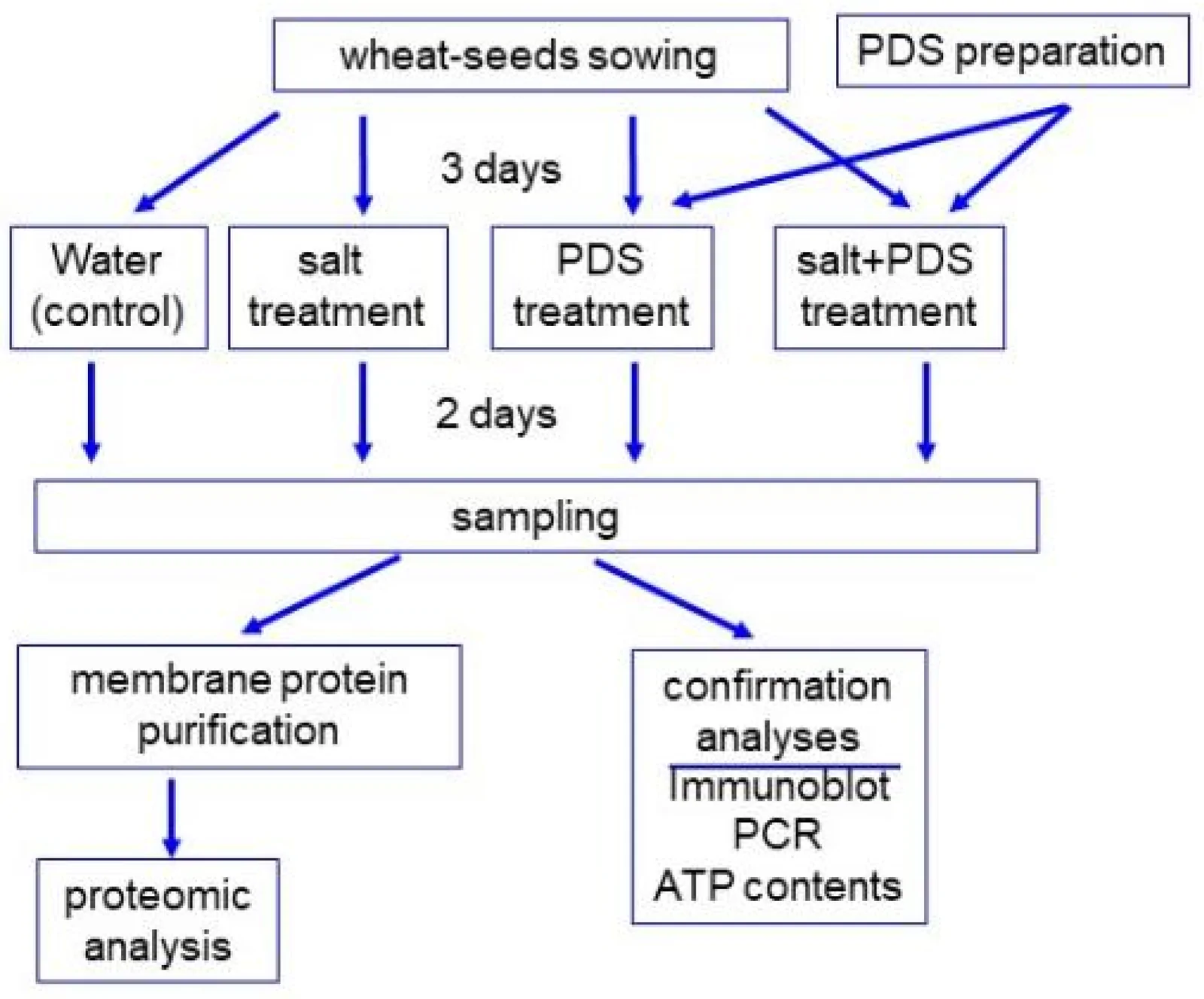
The experimental design for the investigation of the effect of PDS solution on wheat under salt stress. After 3 days of sowing, wheat was treated with or without 2000 ppm PDS solution (PDS) with or without 100 mM NaCl (salt) for 2 days. Membrane proteins in wheat root were purified and proteomic analysis was conducted. For confirmation experiments, immunoblot, qRT-PCR, and ATP-content analyses were performed using the root and leaf of wheat. All experiments were performed with 3 independent biological replicates.

**Figure 3:**
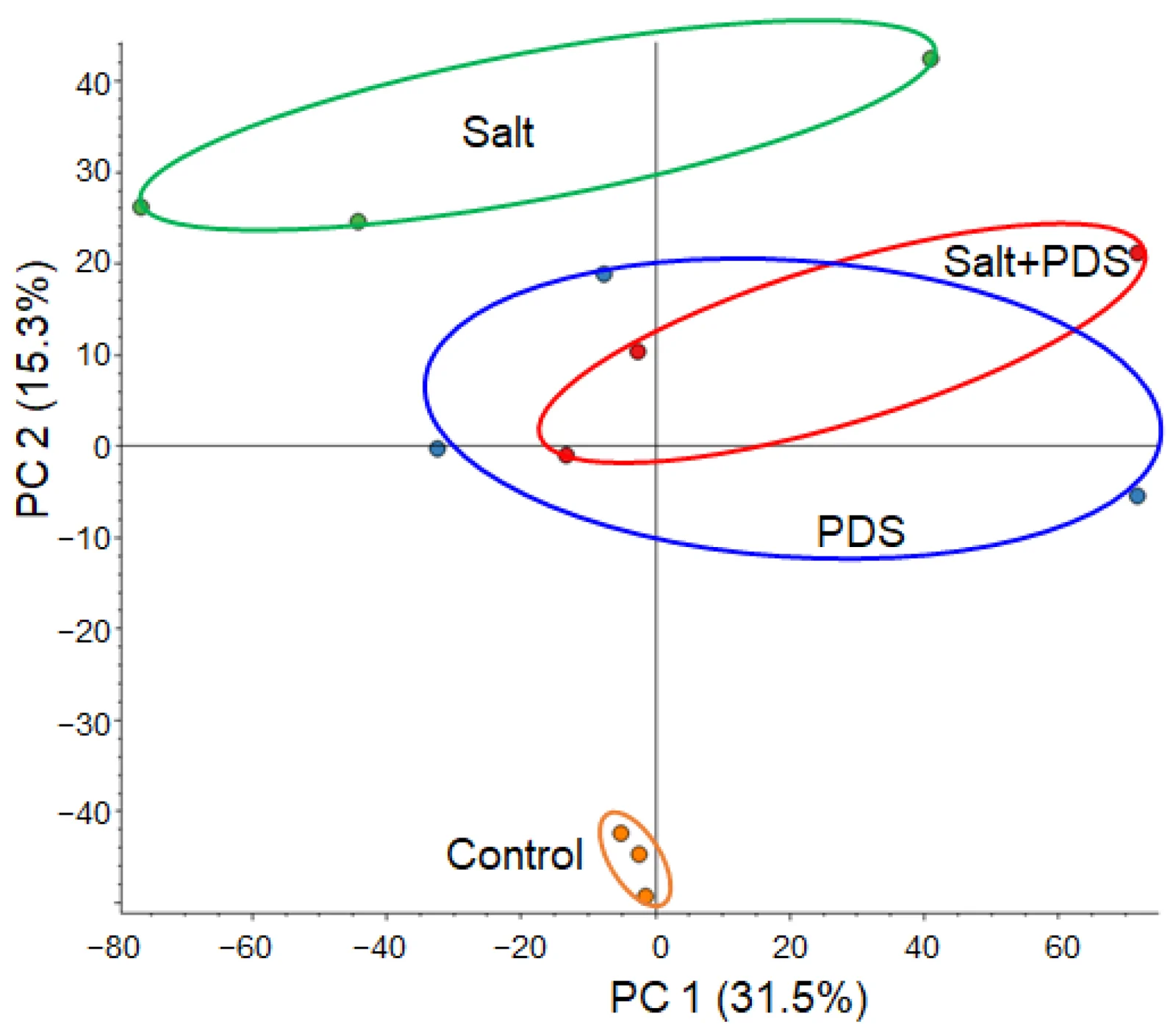
Overview of total proteomic data from 12 samples of wheat root based on principal component analysis. Three-day-old wheat was exposed without or with PDS solution under with or without salt stress for 2 days. Proteomic analysis was performed with 3 independent biological replicates for each treatment. Principal component analysis was performed with Proteome Discoverer.

To investigate the cellular mechanism in wheat growth by the application of PDS solution under salt stress, a gel-free/label-free proteomics was conducted using membrane proteins of wheat roots. A PDS solution with 2000 ppm promoted the growth of wheat inhibited by 100 mM NaCl through the regulation of energy metabolism and flavonoid metabolism [27]. Additionally, a PDS solution with 2000 ppm promoted the growth of wheat during 200 mM NaCl treatment through the alternation of nuclear proteins in a way that contributes to chromatin remodeling and transcription [29]. In the present study, a 2000 ppm PDS solution, whose concentration enhances salt tolerance in wheat, was used. Four kinds of treatments, which were control (untreated), salt (200 mM NaCl), 2000 ppm PDS, and salt+PDS, were performed (Figure 2). Membrane proteins purified from the wheat root after treatment were enriched, reduced, alkylated, and digested. After analysis by LC-MS/MS, the relative abundance of proteins was compared to each other. In total, 6765 proteins were identified by LC-MS/MS analysis. The proteomic results of all 12 samples from the four groups were compared by principal component analysis, which displayed the varied accumulation patterns of proteins from the four different kinds of treatments (Figure 3). This result indicated that the salt stress largely affected the membrane proteins in wheat root; however, this effect was not significant at the protein level with the application of PDS solution even under stress (Figure 3).

**Figure 4:**
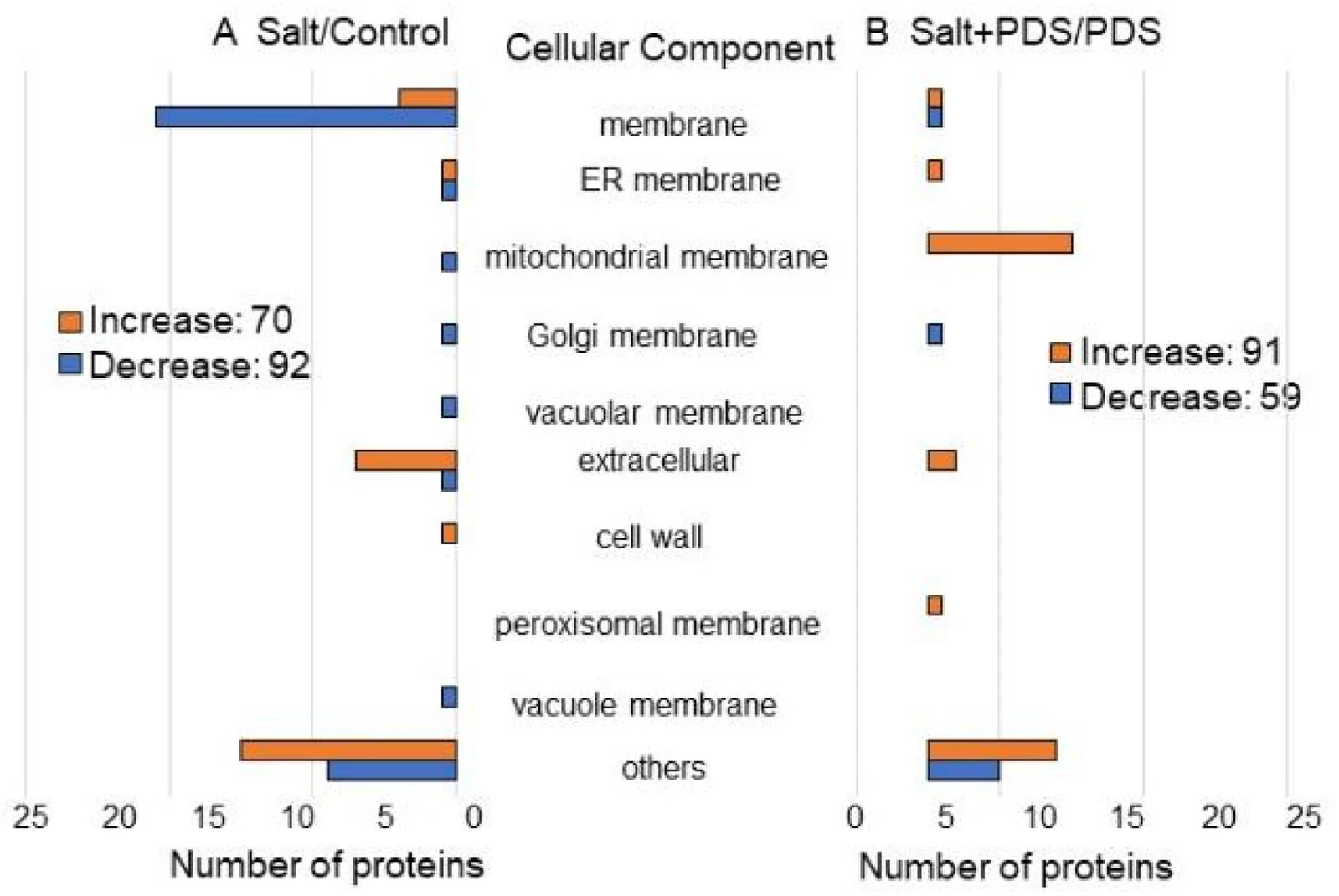
Functional categories of membrane proteins with differential abundance in wheat root with PDS solution under salt stress. Four kinds of treatments, which were control, salt, PDS, salt+PDS, were performed. Membrane proteins purified from wheat root after treatment were enriched, reduced, alkylated, and digested. After analysis by LC-MS/MS, the relative abundance of proteins from wheat was compared as follows: salt/control (**A**) (Table S1) and salt+PDS/PDS (**B**) (Table S2). Functional categories of changed proteins were determined using gene-ontology analysis. Orange and blue columns show increased and decreased proteins, respectively.

The abundance of 162 proteins differentially changed with the *p*-value < 0.05 and fold change >1.5 and/or <2/3 in wheat root under salt stress was compared to the control condition (Table S1). Among the 162 proteins, 70 and 92 proteins increased and decreased, respectively, under salt stress compared to the control condition (Table S1 and Figure 4A). On the other hand, the abundance of another 150 proteins differentially changed with the *p*-value < 0.05 and fold change >1.5 and/or <2/3 in wheat root applied with the PDS solution under salt stress compared to PDS solution alone (Table S2). Among the 150 proteins, 91 and 59 proteins increased and decreased, respectively, with the application of the PDS solution under salt stress compared to PDS solution alone (Table S2 and Figure 4B). The functional category of identified proteins was obtained using gene-ontology analysis (Figure 4). Oppositely changed proteins were associated with the plasma membrane, endoplasmic reticulum membrane, and mitochondrial membrane in cellular components within two groups, which were salt/control and salt+PDS/PDS.

Furthermore, proteins selected in the membrane category were listed (Table 1). Four and 19 proteins increased and decreased by salt stress compared with control, respectively; however, 20 and seven proteins increased and decreased by salt+PDS compared with PDS. Among these proteins, the accumulation of ATP-binding cassette (ABC) transporter decreased by salt stress compared with control; however, it increased by application of PDS solution even if it was under stress. Under salt stress, the increase in ammonium transporters with PDS solution was significant compared to that without PDS solution (Table 1). Results from the proteomic analysis were subsequently confirmed by protein accumulation with immunoblot and RNA expression with qRT-PCR as well as ATP content.

### 2.3. Immunoblot Analysis of Mitochondrial Ascorbate Peroxidase and Other ROS-Scavenging Enzymes in Wheat with Application of PDS Solution Under Salt Stress

**Figure 5:**
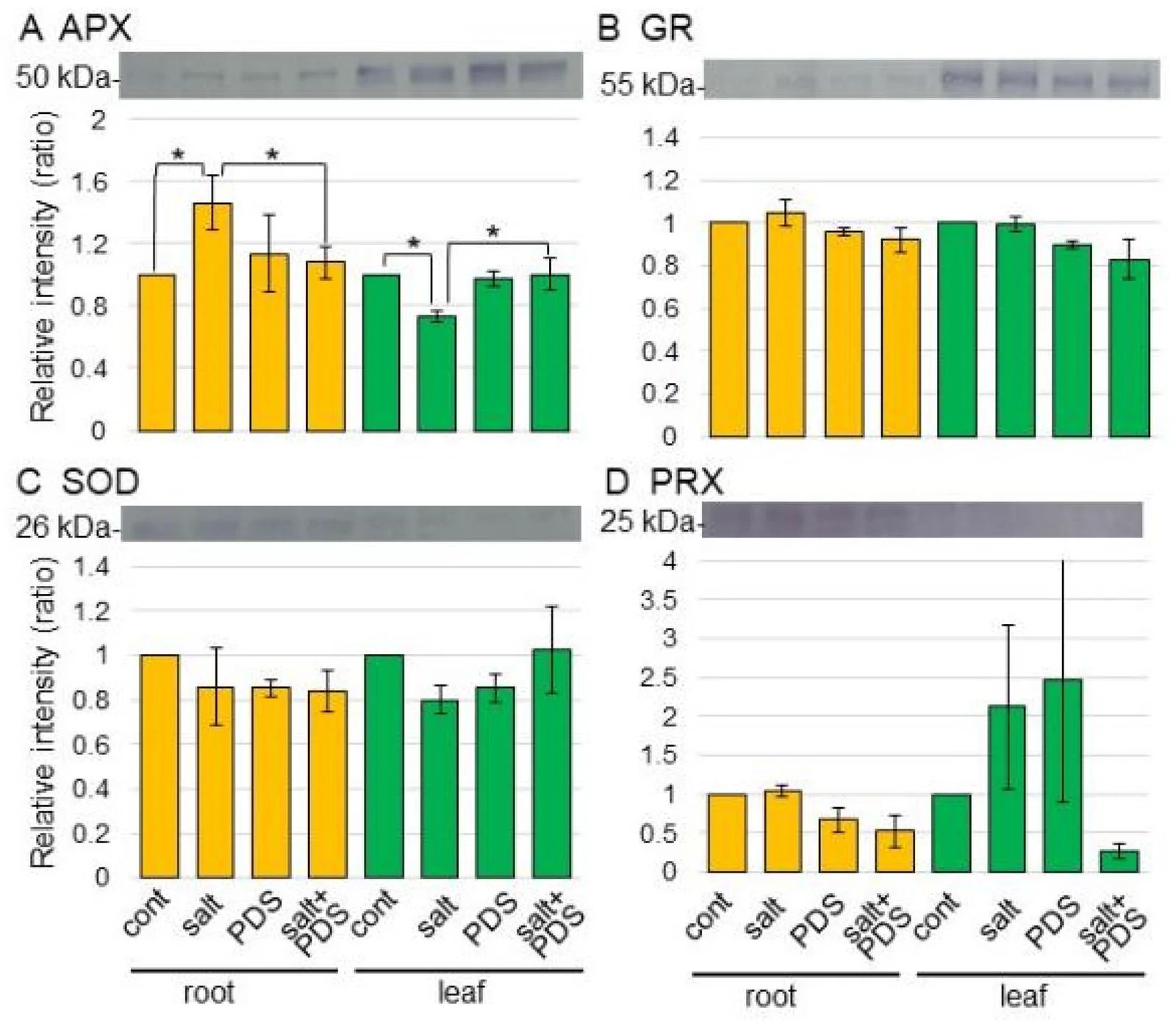
Immunoblot analysis of the proteins involved in wheat treated with PDS solution under salt stress. Proteins extracted from wheat root and leaf were separated on SDS-polyacrylamide gel by electrophoresis and transferred onto membranes. The membranes were cross-reacted with anti-APX (**A**), GR (**B**), SOD (**C**), and PRX (**D**) antibodies. A staining pattern with Coomassie brilliant blue was used as a loading control (Figure S2). The integrated densities of the bands were calculated using ImageJ software. The data are presented as mean ± SD from 3 independent biological replicates (Figures S3–S6). Student’s *t*-test was used to compare values between 2 groups. Asterisks indicate significant changes (* *p* < 0.05).

To better uncover the change in proteins from different treatments, the immunoblot analysis was performed with mitochondrial APX (Figure 5A and Figure S3), glutathione reductase (GR) (Figure 5B and Figure S4), superoxide dismutase (SOD) (Figure 5C and Figure S5), and peroxiredoxin (PRX) (Figure 5D and Figure S6). Proteins extracted from the root and leaf of wheat were separated on the SDS-polyacrylamide gel electrophoresis, and a staining pattern with Coomassie brilliant blue was used as a loading control (Figure S2). The integrated densities of bands were calculated using ImageJ software (version 1.53e, Java1.8.0 172, 64 bit; National Institutes of Health, Bethesda, MD, USA) with triplicated immunoblot results (Figures S3–S6). This analysis confirmed that mitochondrial APX in the root increased with salt stress and decreased with treatment of PDS solution; however, the accumulation of mitochondrial APX in leaf decreased with salt stress and increased with treatment of PDS solution (Figure 5A,B).

### 2.4. ATP Content in Wheat with Application of PDS Solution Under Salt Stress

**Figure 6:**
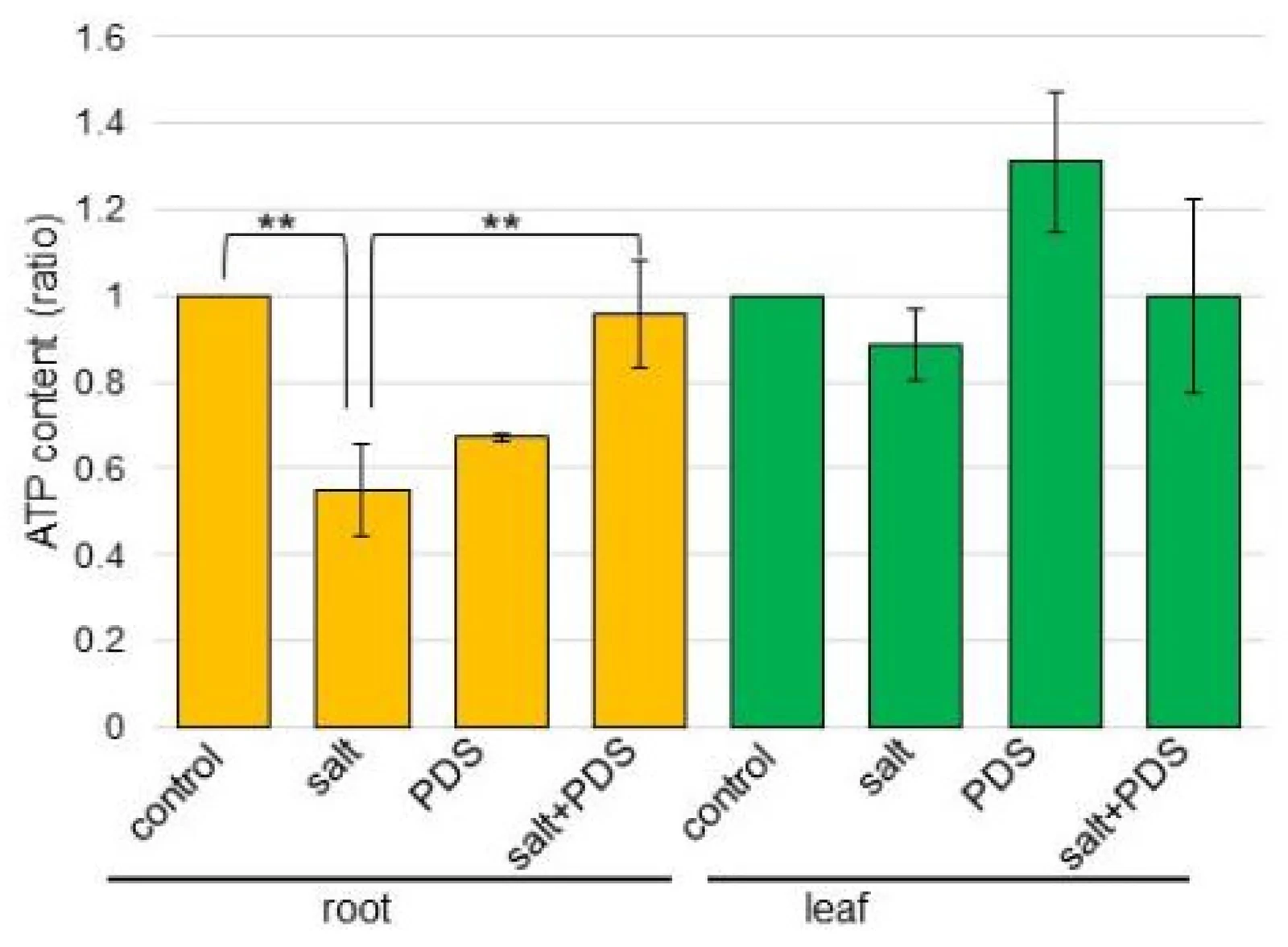
The contents of ATP in wheat treated with the PDS solution under salt stress. Wheat seeds were sown and treated with or without 2000 ppm PDS solution. Three-day-old wheat was treated with or without salt stress for 2 days. Metabolites were extracted from the root and leaf. The ATP contents were measured for each sample. The data are given as the mean ± SD from 3 independent biological replicates. Student’s *t*-test was used to compare values between 2 groups. Asterisks indicate significant changes (** *p* < 0.01).

Because proteins involved in the mitochondrion were oppositely changed in wheat with or without PDS solution under salt stress (Figure 4), the contents of ATP were analyzed (Figure 6). The contents of ATP in root decreased with salt stress and recovered with treatment of PDS solution even if under stress (Figure 6).

### 2.5. Immunoblot Analysis of Membrane-Associated Proteins in Wheat with Application of PDS Solution Under Salt Stress

**Figure 7:**
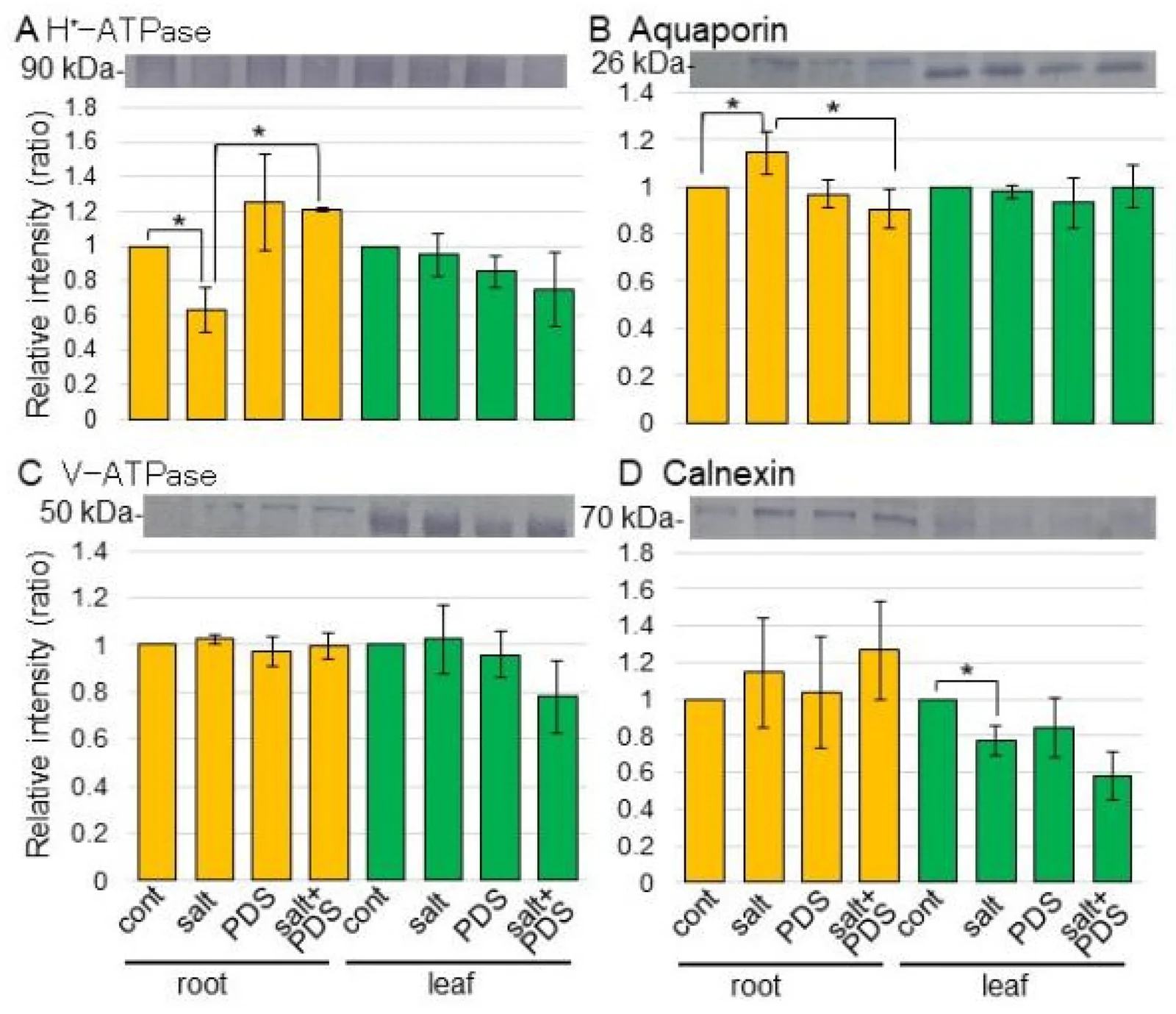
Immunoblot analysis of the proteins involved in wheat treated with PDS solution under salt stress. Proteins extracted from wheat root and leaf were separated on SDS-polyacrylamide gel by electrophoresis and transferred onto membranes. The membranes were cross-reacted with anti-H^+^ATPase (**A**), aquaporin (**B**), V-ATPase (**C**), and Calnexin (**D**) antibodies. A staining pattern with Coomassie brilliant blue was used as a loading control (Figure S2). The integrated densities of the bands were calculated using ImageJ software. The data are presented as mean ± SD from 3 independent biological replicates (Figures S7–S10). Student’s *t*-test was used to compare values between 2 groups. Asterisks indicate significant changes (* *p* < 0.05).

To better uncover the change in membrane proteins from different treatments, immunoblot analysis of H^+^-ATPase, aquaporin, V-ATPase, and calnexin, was performed (Figure 7). Proteins extracted from the root and leaf of wheat were separated on the SDS-polyacrylamide gel by electrophoresis and transferred onto polyvinylidene difluoride (PVDF) membranes. The PVDF membranes were cross-reacted with anti- H^+^-ATPase (Figure 7A), aquaporin (Figure 7B), V-ATPase (Figure 7C), and calnexin (Figure 7D) antibodies. A staining pattern with Coomassie brilliant blue was used as a loading control (Figure S2). The integrated densities of bands were calculated using ImageJ software with triplicated immunoblot results (Figures S7–S10). H^+^-ATPase decreased with salt stress and increased with treatment of PDS solution in the root (Figure 7A); however, aquaporin increased with salt stress and decreased with treatment of PDS solution (Figure 7B). The abundance of V-ATPase and calnexin did not change with any treatments (Figure 7C,D).

### 2.6. qRT-PCR Analysis of Gene Encoding Ammonium Transporter in Wheat with Application of PDS Solution Under Salt Stress

**Figure 8:**
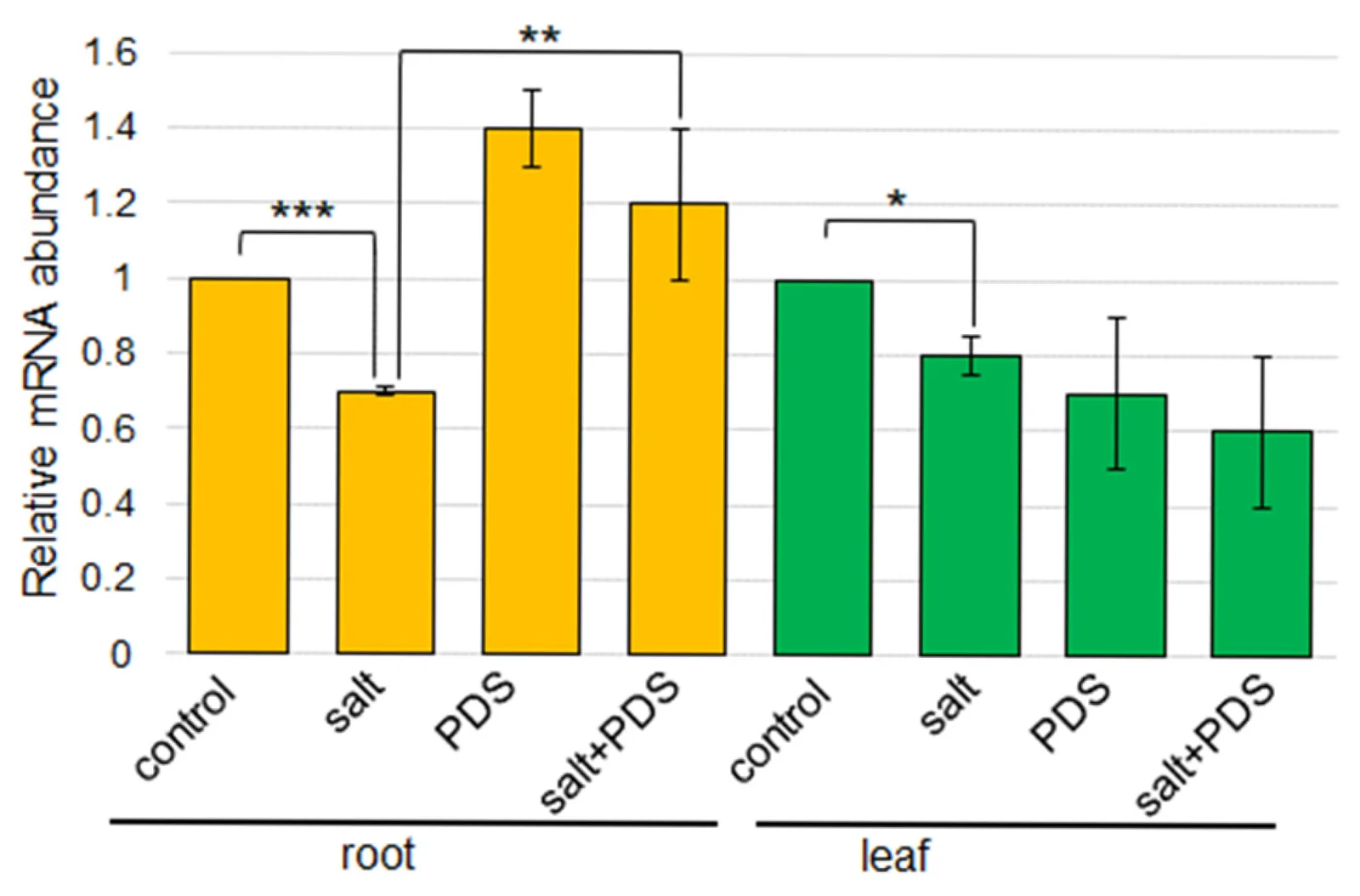
Gene expression of *ammonium transporter* in wheat root and leaf treated with PDS solution under salt stress. After extraction of total RNA from root and leaf, gene-expression analysis of *ammonium transporter* was performed using qRT-PCR. *18S rRNA* was used as an internal control. Data are shown as the means ± SD from 3 independent biological replicates. Student’s *t*-test was used to compare values between 2 groups. Asterisks indicate significant changes (* *p* < 0.05; ** *p* < 0.01 and *** *p* < 0.001).

Ammonium transporter increased with salt stress and further increased with the application of PDS solution under stress (Table 1). Based on the proteomic results, the change in the expression level of the gene, which encodes ammonium transporter in response against salt stress with PDS solution was confirmed with qRT-PCR analysis (Figure 8). *Ammonium transporter*-specific oligonucleotide was used to amplify transcripts of the total RNA isolated from the wheat root and leaf (Figure 8). The expression level of *18S rRNA* was used as the internal control. The expression of *ammonium transporter* was significantly downregulated in the root and leaf by salt stress. However, the expression of this gene was upregulated in the root with treatment of PDS solution, even with stress (Figure 8).

## 3. Discussion

### 3.1. PDS Solution Acts on Membrane Proteins in Wheat Roots Under Salt Stress

PDS solution was reported to promote wheat growth under salt stress through the regulation of energy and flavonoid metabolism [27]. In contrast, in soybean roots, PDS solution affects membrane and nuclear proteins, thereby influencing transcriptional activation and energy production [28]. Under Cd stress conditions, the application of PDS solution effectively increased chlorophyll content, relative water content, stomatal conductance, and total phenolic content, while significantly reducing malondialdehyde levels and membrane damage [23]. Phytohormones produced following the application of PDS solution have been shown to enhance chlorophyll content, soluble sugar content, relative water content, and membrane stability in wheat [30]. Furthermore, phytohormones suppress the generation of reactive oxygen species, such as superoxide and hydroxyl radicals, which damage membrane lipids, and promote their scavenging [31]. It was also reported that treatment with putrescine and benzyladenine enhances membrane stability in wheat [32]. Based on the results of the present study together with previous findings, PDS solution is suggested to act on cellular membranes in wheat under salt stress, thereby improving membrane stability and contributing to the induction of salt-stress tolerance.

This study employed membrane proteomics to investigate the mechanism by which PDS solution promotes root growth in wheat under salt stress. Based on immunoblot analysis using subcellular marker proteins [33], the membrane fraction obtained in the present study was confirmed to be highly enriched in membrane proteins (Figure 1). Using this purification protocol, membrane proteomic analysis was conducted. Principal component analysis (Figure 3) indicated that salt stress markedly altered membrane-protein profiles in wheat roots, whereas these changes are partially restored by PDS-solution treatment even under stress conditions. As this proteomics is exploratory in nature, proteins identified using an unadjusted *p*-value should be considered as candidate proteins. Given the possibility of false positives, selected candidates were further validated by immunoblot analysis, gene-expression analysis, and ATP-content measurement. Among these candidates, an ABC transporter was identified, whose abundance decreased under salt stress but increased upon PDS-solution treatment (Table 1). ABC transporters are a large and diverse family of membrane proteins, which utilize ATP hydrolysis to transport various compounds across membranes against concentration gradients [34]. In particular, members of the *ABCG* subfamily were implicated in tolerance to multiple abiotic stresses [35]. Furthermore, previous studies have shown that activation of hormone signaling pathways can regulate ABC transporter-related genes in wheat roots [36]. Taken together, these results suggest that PDS solution modulates membrane proteins in wheat roots, contributing to enhanced salt tolerance.

### 3.2. The Accumulation of APX Due to Salt Stress Is Reduced by the Addition of PDS Solution

Under non-stress conditions, intracellular ROS levels are tightly regulated by a sophisticated antioxidant defense system [37]. However, under abiotic stress, this balance between ROS production and scavenging is disrupted, leading to a rapid accumulation of ROS [38]. Although ROS function as important signaling molecules in plants, their excessive accumulation can cause oxidative damage to biomolecules, including lipid peroxidation and loss of membrane integrity [39]. Salt stress has been shown to rapidly induce ROS production [40], and mitigating oxidative damage is therefore a key component of salt tolerance in plants [41]. These findings suggest that protection against ROS-induced damage to membrane components, including membrane proteins, is closely associated with stress tolerance. In the present study, APX levels in wheat roots increased under salt stress but decreased following PDS-solution treatment (Figure 5). APX is a key ROS-scavenging enzyme whose expression and activity are regulated in response to environmental stress and changes in cellular redox status [42,43]. Previous studies have suggested that antioxidant enzyme induction is closely associated with ROS accumulation under abiotic stress conditions [44]. Taken together with these reports, these findings suggest that PDS solution may alleviate oxidative stress, thereby reducing the requirement for APX-mediated ROS scavenging and contributing to the protection of membrane proteins under salt stress.

### 3.3. H^+^-ATPase and Aquaporin Are Involved in Salt-Stress Tolerance Induced by PDS Solution

Water transport across the plasma and vacuolar membranes plays a central role in osmoregulation and the maintenance of turgor pressure in plant cells, which is essential for cell expansion, growth, and stomatal function [45,46]. Plasma membrane H^+^-ATPases provide the energy required for ion transport, thereby facilitating water movement across membranes and contributing to the generation of turgor pressure needed for cell expansion [47]. These enzymes are regulated by hormonal signaling and are involved in plant responses to both biotic and abiotic stresses [48]. Under abiotic stress conditions, including those mediated by abscisic acid, H^+^-ATPase activity is often downregulated, promoting stomatal closure and reducing water loss [47,49]. Aquaporins are major water channels that govern cellular water permeability and play a key role in regulating water flux, turgor, and osmoregulation in plant cells [50]. Their activity is controlled by both protein abundance and channel conductance, allowing precise regulation of water transport under varying environmental conditions [51,52]. In the present study, H^+^-ATPase levels decreased under salt stress but increased following PDS treatment, whereas aquaporin levels showed the opposite trend (Figure 7). Taken together with previous studies, these findings suggest that the modulation of membrane proteins involved in water and ion transport may be one of the mechanisms underlying PDS solution-induced adaptation of wheat roots to salt stress.

The passive influx of Na^+^ into the cytoplasm of root epidermal and cortical cells from soil [53] occurs via *Arabidopsis* PIP2;1 and PIP2;2 aquaporins, which are abundant in the plasma membrane of root epidermal cells and are permeable to Na^+^ [54]. In the current study, high-concentration NaCl increased the abundance of aquaporin (Figure 7). The ability of plants to exclude cytosolic Na^+^ is strongly correlated with salt-stress tolerance in various plant species. This exclusion is mediated by plasma membrane Na^+^/H^+^ antiporter encoded by the *SOS1* (*Salt Overly Sensitive*) gene and driven by a proton gradient generated with plasma membrane H^+^-ATPase [55], which involves energy-dependent pathways fueled by H^+^-ATPase [56,57]. In the current study, high-concentration NaCl decreased the abundance of H^+^-ATPase (Figure 7). The coordinated activity of H^+^-ATPase and aquaporins enhances the transport of water and ions across the plasma membrane in response to physiological, developmental, and adaptive biological as well as abiotic stresses in plants [47,58]. The plasma membrane H^+^-ATPase AHA1 and the aquaporins PIP2;1/PIP2;2 associated within a SYP132 (SYNTAXIN OF PLANTS 132)-complex, which is sensitive to abiotic stress [58]. These reports and the current result indicate that H^+^-ATPases and aquaporins on the plasma membrane regulate water and proton transport in order for PDS solution to alleviate salt stress on wheat roots.

### 3.4. The Suppression of Ammonium-Transporter Expression Due to Salt Stress Is Restored by PDS Solution

The accumulation of ionic components, such as K^+^, Na^+^, and Cl^−^, disturbs ionic homeostasis, leading to cell membrane dysfunction, decreased metabolic activity, and a further reduction in plant growth [59]. High-affinity potassium transporters, such as *HAK1*, are essential for maintaining cellular K^+^ homeostasis, which is crucial for plant growth under both normal and stressful conditions [60]. Wheat *HAK1* alleviates salt stress by regulating ROS levels, maintaining K^+^/Na^+^ homeostasis, and upregulating auxin signaling-related genes [61]. In our proteomic analysis, the abundance of potassium transporters decreased under salt stress regardless of PDS-solution treatment (Table 1). This suggests that other mechanisms may contribute to the salt-stress tolerance induced by PDS solution. Ammonium uptake through ammonium transporters plays a critical role in meeting plant nitrogen requirements, particularly in flooded or acidic soils where ammonium concentrations are high [62]. In *Arabidopsis*, root ammonium uptake is mediated by ammonium transporters belonging to the AMT1 and AMT2 subfamilies [63]. Previous studies have shown that *AMT1* expression is reduced under salt-stress conditions [64]. In the present study, an ammonium transporter that was downregulated by salt stress was upregulated following PDS-solution treatment (Figure 8). Taken together with previous reports, these findings suggest that the regulation of ammonium transporters may represent one of the mechanisms underlying PDS solution-induced salt-stress tolerance in wheat roots.

## 4. Materials and Methods

### 4.1. Isolation and Purity Check of Membrane Fraction

#### 4.1.1. Isolation of Membrane Fraction

All isolation procedures were performed on ice. Membranes were fractionated according to the instructions of the Mem-PER Plus Membrane Protein Extraction Kit (Thermo Fisher Scientific, San Jose, CA, USA) provided by the manufacturer with some modifications. The procedures were described in the previous study [33]. Briefly, a portion (2.0 g) of samples was ground in 1 mL of permeabilization buffer using a mortar and pestle. The homogenate was transferred to a tube, incubated for 10 min at 4 °C with constant mixing, and centrifuged at 16,000× *g* for 15 min at 4 °C to pellet the permeabilized cells. The supernatant containing cytosolic proteins was transferred to a new tube. The pellet was resuspended in 0.5 mL of solubilization buffer with pipetting, and a homogeneous suspension was incubated for 30 min at 4 °C with constant mixing. After centrifugation at 16,000× *g* for 15 min at 4 °C, the supernatant containing solubilized membrane and membrane-associated proteins was sonicated with lysis buffer consisting of 7 M urea, 2 M thiourea, 5% CHAPS, and 2 mM tributylphosphine. After sonication, the homogenate was centrifuged at 12,000× *g* for 30 min at 4 °C, and the supernatant was collected as membrane proteins. Protein concentration was analyzed using XL-Bradford solution (Integrale, Naruto, Japan). After mixing and incubation for 5 min, the absorbance was measured at 595 nm and determined with bovine-serum albumin as the standard [65].

#### 4.1.2. Purity Check Using Immunoblot Analysis

SDS-sample buffer consisting of 60 mM Tris-HCl (pH 6.8), 2% SDS, 10% glycerol, and 5% dithiothreitol was added to membrane proteins [66]. Quantified proteins (10 µg) were separated by electrophoresis on a 10% SDS-polyacrylamide gel and transferred onto PVDF membrane using a semidry transfer blotter [33]. The blotted PVDF membrane was blocked for 5 min in Bullet Blocking One reagent (Nacalai Tesque, Kyoto, Japan) and cross-reacted with the primary antibodies for 30 min. As the primary antibodies, anti-H^+^-ATPase (Agrisera, Vannas, Sweden), anti-calnexin [67], and anti-APX [68] were used. Anti-rabbit IgG conjugated with horseradish peroxidase (BioRad, Hercules, CA, USA) was used as the secondary antibody. After 30 min incubation, signals were detected using 3,3′,5,5′-tetramethylbenzidine solution (Nacarai Tesque) for 1–10 min. The integrated densities of bands were calculated using ImageJ software version 1.53e, Java1.8.0 172, 64 bit; National Institutes of Health, Bethesda, MD, USA).

### 4.2. Preparation of PDS Solution and Its Application to Wheat

#### 4.2.1. Preparation of PDS Solution

PDS solution was prepared from *Cymbopogon* jwarancusa (Kohat University of Science and Technology, Kohat, Pakistan) [69], whose protocol was modified from previous methods [70]. Samples were washed with water to remove the dust particles and were shade-dried. A portion (333 g) of semi-dried plant was smoldered in an airtight furnace. Smoke was bubbled through 1 L of distilled water in a beaker to gain concentrated smoke solution, which was filtered through filter paper. The 1,000,000 ppm concentrated PDS solution was diluted 500-fold with water and used at 2000 ppm.

#### 4.2.2. Application of PDS Solution to Wheat

Seeds of wheat (*Triticum aestivum* L. cultivar Nourin 61) were sown in silica sand, which was placed in a seedling case. Three days later, wheat seedlings were treated with or without 2000 ppm PDS solution, as well as with or without 200 mM NaCl. These treatments were incorporated into the sand. Seedlings were maintained at 25 °C in a growth chamber illuminated with white-fluorescent light (200 µmol m^−2^ s^−1^, 16 h light period/day). Root was used for proteomic analysis. Furthermore, root and leaf were used in other biological analyses. More than 3 independent experiments were performed as biological replicates for all experiments. Seeds were sown with independent biological replicates on different days.

### 4.3. Proteomic Analysis

#### 4.3.1. Protein Enrichment, Reduction, Alkylation, and Digestion

Quantified membrane proteins (100 µg) were adjusted to a final volume of 100 µL. Proteins were enriched, reduced, alkylated, and digested using previous methods [71]. Briefly, methanol (400 µL) was added to each sample followed by 100 µL of chloroform and 300 µL of water. After centrifugation at 16,000× *g* for 10 min to achieve phase separation, the upper phase was discarded and 300 µL of methanol was added to the lower phase. After centrifugation at 16,000× *g* for 10 min, the pellet was collected as the soluble fraction. Proteins were resuspended in 50 mM ammonium bicarbonate, reduced with 50 mM dithiothreitol for 30 min at 56 °C, and alkylated with 50 mM iodoacetamide for 30 min at 37 °C in the dark. After incubation, proteins were digested with trypsin and lysyl endopeptidase (FUJIFILM Wako Pure Chemical, Osaka, Japan) at a 1:100 enzyme/protein ratio for 16 h at 37 °C. Peptides were desalted with a MonoSpin C18 Column (GL Sciences, Tokyo, Japan) and acidified with 1% trifluoroacetic acid.

#### 4.3.2. Protein Identification Using NanoLC-MS/MS

The conditions of LC (EASY-nLC 1000; Thermo Fisher Scientific) and MS (Orbitrap Fusion ETD MS; Thermo Fisher Scientific) were described in the previous study [72]. The peptides were loaded onto the LC system equipped with a trap column (Acclaim PepMap 100 C18 LC column, 3 µm, 75 µm ID × 20 mm; Thermo Fisher Scientific), equilibrated with 0.1% formic acid, and eluted with a linear acetonitrile gradient (0–35%) in 0.1% formic acid at a flow rate of 300 nL min^−1^. The eluted peptides were loaded and separated on the column (EASY-Spray C18 LC column, 3 µm, 75 µm ID × 150 mm; Thermo Fisher Scientific) with a spray voltage of 2 kV (Ion Transfer Tube temperature: 275 °C). The peptide ions were detected using MS in the data-dependent acquisition mode with the installed Xcalibur software (version 4.7.69.37; Thermo Fisher Scientific). Full-scan mass spectra were acquired in the MS over 375–1500 *m*/*z* with a resolution of 120,000. The most potent precursor ions were picked for collision-induced fragmentation in the linear ion trap with a normalized collision energy of 35%. In order to stop the repeated selection of peptides, dynamic exclusion was used within 60 s.

#### 4.3.3. MS Data Analysis

The MS/MS searches were carried out using MASCOT (version 2.6.2; Matrix Science, London, UK) and SEQUEST HT search algorithms against the UniprotKB *Triticum aestivum* (Wheat4565_SwissProt_TreEMBL_TaxID4546_CanonicalIsoform; version 20240327) using Proteome Discoverer (version 2.4.1.15; Thermo Fisher Scientific). The workflow was described in the previous study [69]. Spectrum files RC, spectrum selector, MASCOT, SEQUEST HT search nodes, percolator, ptmRS, and minor feature detector nodes were part of both algorithms. Methionine oxidation was considered as a variable modification, while cysteine carbamidomethylation was regarded as a fixed modification. The MS and MS/MS mass tolerances were set at 10 ppm and 0.6 Da, respectively. Trypsin was designated as a protease, and a maximum of 2 missed cleavages was allowed. The false discovery rate was calculated using target-decoy database searches, which was set at 1% for peptide identification.

#### 4.3.4. Differential Analysis of Proteins Using MS Data

Label-free quantification was performed with Proteome Discoverer using precursor-ions quantifier nodes. No additional sample-wise normalization was applied at this stage. Principal component analysis was also conducted in Proteome Discoverer. Downstream statistical analyses were performed using Perseus (version 1.6.15.0) [73]. Protein abundances were log_2_-transformed. To reduce systematic global differences in signal intensity across samples, global median centering was applied by subtracting the median value of each sample, assuming that the majority of proteins are not differentially expressed. Each group consisted of three biological replicates, and proteins were required to have at least three valid values per group. Missing values were imputed from a normal distribution (width = 0.3, down shift = 1.8). Statistical significance was assessed using Student’s *t*-test. Functional annotation of differentially abundant proteins was performed using the gene-ontology database (http://www.geneontology.org/; accessed on 10 September 2024).

### 4.4. Protein Extraction and Immunoblot Analysis

#### 4.4.1. Protein Extraction

A portion (500 mg) of samples was ground in 500 µL of lysis buffer consisting of 50 mM Tris-HCl (pH 7.6), 100 mM NaCl, 1% Nonidet-P40, 0.1% SDS, and protease inhibitor (Nacalai Tesque, Kyoto, Japan) into a mortar and pestle on ice. The suspension was centrifuged twice at 16,000× *g* for 10 min at 4 °C and supernatant was used as crude protein extract. The protein concentration measurement method is the same as in “Section 4.1.1”.

#### 4.4.2. Immunoblot Analysis

The immunoblot analysis method was the same as in “Section 4.1.2”. As the primary antibodies, anti-APX (see “Section 4.1.2”), anti-GR (Agrisera), anti-SOD (Proteintech, Rosemont, IL, USA), anti-PRX [74], anti-H^+^-ATPase (see “Section 4.1.2”), anti-calnexin (see “Section 4.1.2”), anti-aquaporin (Cosmo Bio, Tokyo, Japan), and anti-V-ATPase (Cosmo Bio) were used.

### 4.5. RNA Extraction and PCR Analysis

A portion (500 mg) of samples was snap-frozen in liquid nitrogen and ground into a powder using a mortar and pestle. Total RNA was isolated with the RNeasy Plant Mini Kit (Qiagen, Venlo, The Netherlands) according to the protocol from the manufacturer. First-strand cDNA was synthesized from 1 μg of total RNA using the iSuperscript Reverse Transcription Supermix (Bio-Rad). Gene-specific primers were constructed with Primer3Plus software (https://www.bioinformatics.nl/cgi-bin/primer3plus/primer3plus.cgi/; accessed on 1 June 2026) [75] and used to amplify the 200–300 bp regions. Gene-specific primers for 18S rRNA (X02623) (F 5′-TGATTAACAGGGACAGTCGG-3′; R 5′-ACGGTATCTGATCGTCTTCG-3′) and ammonium transporter (A0A3B6NPI0) (F 5′- CGGCTTCGACTACAGCTTCT -3′; R 5′- GGACTTGAGGATGGTGAGGA -3′) were synthesized. A reaction mixture (20 μL) was used to perform qRT-PCR using SYBR Green Supermix (Invitrogen, Waltham, MA, USA). The PCR conditions included an initial step for 30 s at 95 °C, followed by 40 cycles of 10 s at 95 °C, and for 30 s at 60 °C. Gene expression was normalized using *18S rRNA* as an internal control. Relative expression was calculated according to the 2^−ΔΔct^ method.

### 4.6. Measurement of ATP Content

A portion (125 mg) of samples was homogenized in 250 µL of the ATP assay buffer and centrifuged at 16,000× *g* for 10 min at 4 °C. For sample deproteinization and neutralization, the supernatant was treated with a Deproteinizing Sample Preparation Kit (Biovision, Milpitas, CA, USA) [27]. Extracts (50 µL) were added to 50 µL of a reaction mixture consisting of an ATP converter, ATP probe, ATP developer, and ATP assay buffer in an ATP Colorimetric/Fluorometric Assay Kit (Biovision). After mixing and incubation for 30 min at 25 °C in the dark, the absorbance was measured at 570 nm and determined with ATP as the standard.

### 4.7. Statistical Analysis

The statistical significance of data between the 2 groups was analyzed using Student’s *t*-test. A *p*-value of less than 0.05 was considered statistically significant.

## 5. Conclusions

To investigate the salt-tolerant mechanism of wheat caused by PDS solution, membrane proteomics was conducted. Principal component analysis indicated that 200 mM NaCl largely affected the membrane proteins in wheat root; however, this effect is recovered at the membrane-protein level with the application of 2000 ppm PDS solution even under stress. The main findings of this research are as follows: (i) mitochondrial APX in the root increased with salt stress and decreased with treatment of PDS solution; however, its accumulation in leaf decreased with salt stress and increased with treatment of PDS solution; (ii) ATP content decreased with salt stress and increased with treatment of PDS solution even under stress; (iii) H^+^-ATPase accumulation decreased with salt stress and recovered to the control level with treatment of PDS solution; (iv) aquaporin accumulation increased with salt stress and recovered to the control level with treatment of PDS solution; and (v) *ammonium transporter* was significantly downregulated by salt stress; however, its expression was upregulated with treatment of PDS solution. These results suggest that PDS solution may confer salt-stress tolerance to wheat by regulating energy metabolism, water permeability, and ammonium uptake in root membranes.

## Figures and Tables

**Table 1:** List of proteins classified under the “membrane” category within the Cellular Component section of Figure 4.

No	ID	Deference	Protein Name
Salt/Control			
1	A0A3B6NPI0	0.95	Ammonium transporter
2	A0A3B6LPZ8	0.86	L-gulonolactone oxidase
3	A0A3B6CAF8	0.72	Major facilitator superfamily (MFS) profile
4	A0A3B6ILE0	0.68	Protein kinase
5	A0A3B6FSE5	−0.59	Cytochrome P450
6	A0A3B6AWE0	−0.60	TPT
7	A0A3B5Y122	−0.68	Protein kinase
8	A0A3B5YXI2	−0.68	Methyltransferase
9	A0A3B5Z3B3	−0.68	ABC transporter
10	A0A3B6QKD6	−0.68	Transmembrane 9 superfamily member
11	A0A3B6GXZ9	−0.73	Glycosylphosphatidylinositol anchor attachment 1
12	A0A3B5Y5H7	−0.75	ABC transporter
13	A0A3B6NY95	−0.76	Major facilitator superfamily (MFS) profile
14	A0A3B6NIY4	−0.85	P450
15	A0A3B6LWW3	−0.90	Trafficking protein particle complex subunit
16	A0A3B6KDL5	−0.91	Uncharacterized protein
17	A0A1D5UIS5	−1.02	Potassium transporter
18	A0A3B6I5B5	−1.09	Potassium transporter
19	A0A3B6RP28	−1.09	Phospholipase D
20	A0A3B6NLB0	−1.14	Chloride channel protein
21	A0A3B6I3P5	−1.25	Beta-fructofuranosidase
22	A0A3B6JHJ0	−1.29	ATP synthase subunit alpha
23	A0A3B6TKA4	−1.56	Nitrate transmembrane transporter
Salt+PDS/PDS			
1	A0A3B5YR57	2.88	Phospholipase D
2	A0A3B6SMF0	2.15	CNNM transmembrane
3	A0A3B6FZV7	1.89	Protein kinase
4	A0A3B6NPI0	1.51	Ammonium transporter
5	A0A3B6E9Z1	1.33	Uncharacterized protein
6	A0A3B6TN09	1.29	ABC transporter
7	A0A3B6A1A0	1.20	3-deoxy-manno-octulosonate cytidylyltransferase
8	A0A3B6EJN2	1.03	VTT
9	A0A3B6FS48	0.91	MDR-like ABC transporter
10	A0A3B6A243	0.77	t-SNARE coiled-coil homology
11	A0A3B6NNY2	0.75	High-affinity nitrate transporter
12	A0A3B6LEI0	0.74	Mce/MlaD domain-containing protein
13	A0A3B6MU99	0.72	ER membrane protein complex subunit 3
14	A0A3B6NQP9	0.72	Prohibitin
15	A0A3B6B3V5	0.67	Non-specific serine/threonine protein kinase
16	A0A3B6C9J8	0.66	Ammonium transporter
17	A0A0E3IDZ3	0.65	H(+)/Pi cotransporter
18	A0A3B6HWC9	0.63	3-beta hydroxysteroid dehydrogenase/isomerase
19	A0A3B6PTB2	0.62	H(+)-exporting diphosphatase
20	A0A3B6B1E4	0.59	Prohibitin
21	A0A3B6IYZ8	−0.59	Methyltransferase
22	A0A3B6I5B5	−0.92	Potassium transporter
23	A0A1D5UIS5	−1.13	Potassium transporter
24	A0A3B6JHJ0	−1.14	ATP synthase subunit alpha
25	A0A3B6I3P5	−1.83	Beta-fructofuranosidase
26	A0A3B6SDT8	−1.83	L-gulonolactone oxidase
27	A0A3B6SH80	−3.36	Glycosyltransferase 61 catalytic

## Data Availability

MS data, RAW data, peak lists, and result files of proteomic analysis have been deposited in the ProteomeXchange Consortium [76] via the jPOST [77] partner repository under data-set identifier PXD056671.

## Supplementary Materials

The following supporting information can be downloaded at: https://www.mdpi.com/article/10.3390/ijms27167344/s1.

## Abbreviations

The following abbreviations are used in this manuscript:

ABC	ATP-binding cassette
APX	Ascorbate peroxidases
GR	Glutathione reductase
LC	Liquid chromatography
MS	Mass spectrometry
PDS	Plant-derived smoke
PRX	Peroxiredoxin
ROS	Reactive oxygen species
SOD	Superoxide dismutase
qRT-PCR	Quantitative real-time polymerase-chain reaction
