# Supplementary material for: Membrane Proteomics Reveals the Role of Plant-Derived Smoke Solution on Wheat Under Salt Stress
Source: Int J Mol Sci. 2026 Aug 17;27(16):7344. doi: 10.3390/ijms27167344 (PMC13513836; doi:10.3390/ijms27167344)
Supplement: Supplementary file 1 [file ijms-27-07344-s001.zip › Supplemental Figures.pdf]

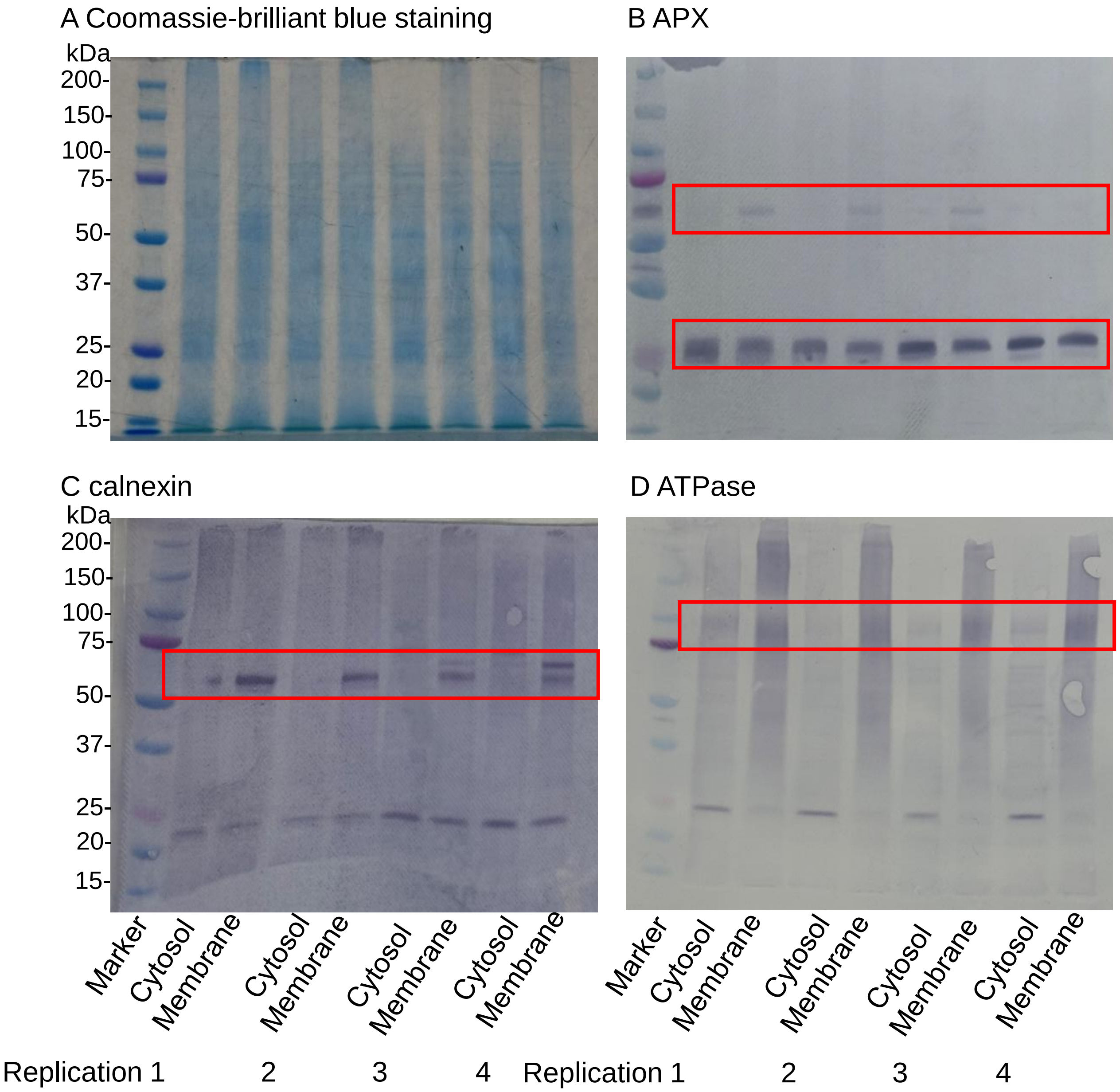

Figure S1. The Coomassie-brilliant blue staining pattern and immunoblot of membrane proteins, which used in Figure 1. Experiments were performed with 4 biological replicates. Quantified proteins (10 µg) from roots and leaves were separated by electrophoresis on a 10% SDS-polyacrylamide. Coomassie-brilliant blue staining was used as a loading control (A). Immunoblots show patterns reacted with anti APX (B), calnexin (C), and ATPase (D) antibodies.

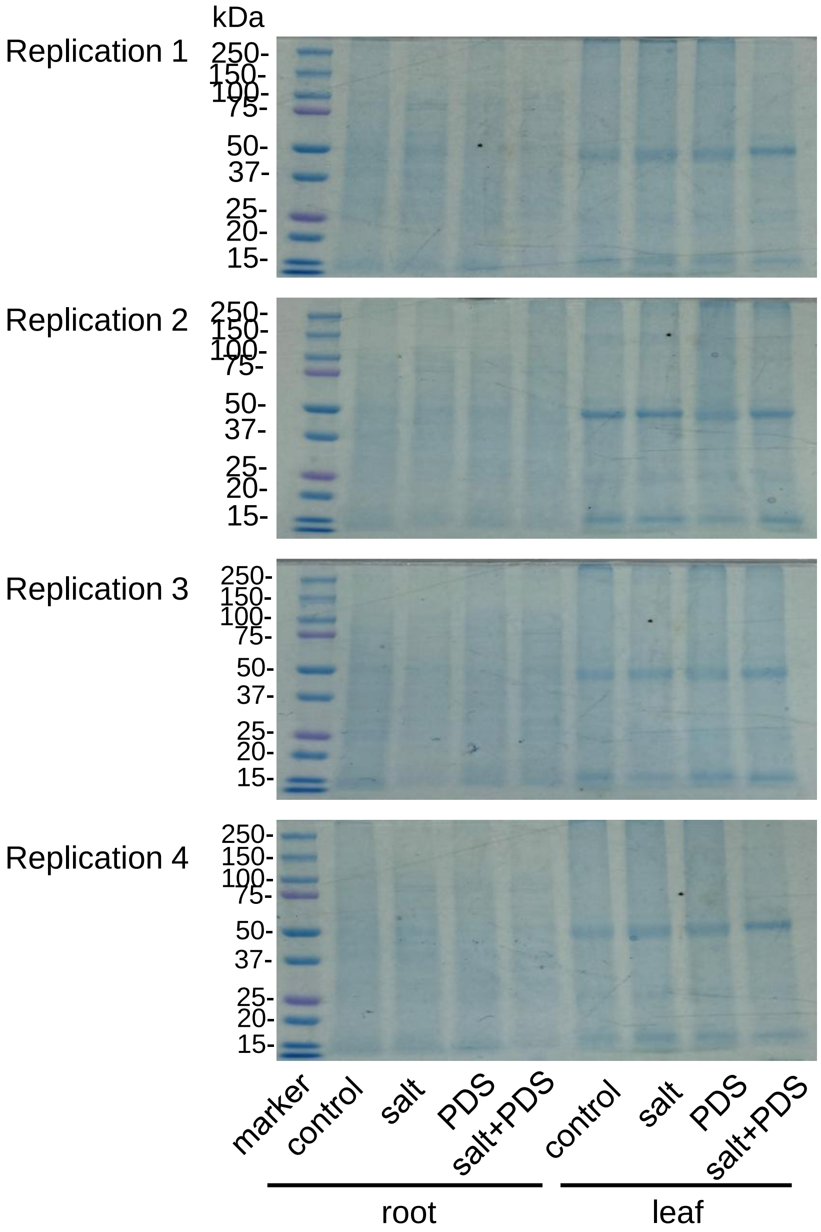

Figure S2. The Coomassie-brilliant blue staining pattern of proteins used for immunoblot analysis.

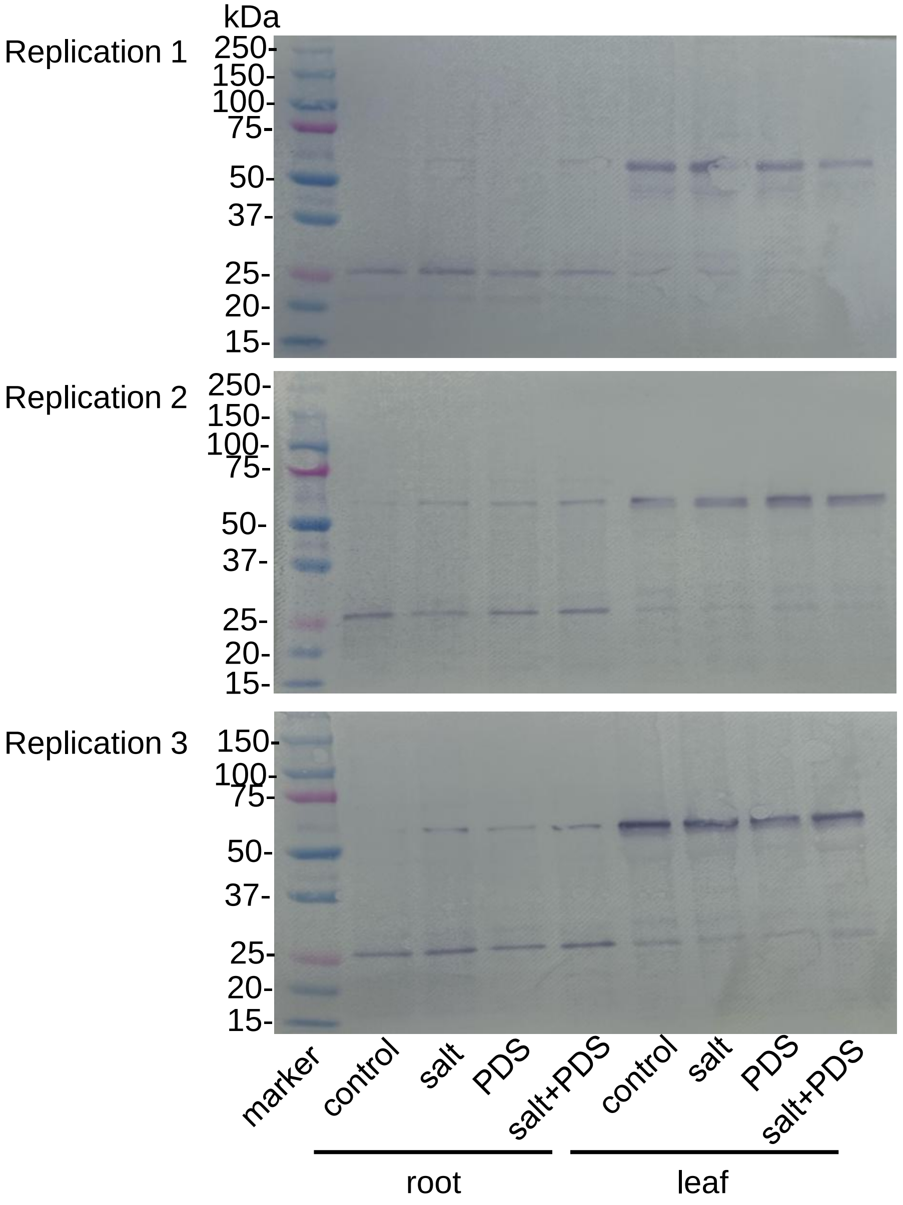

Figure S3. Blots of the entire membrane with anti-APX antibody, which were used in Figure 5A.

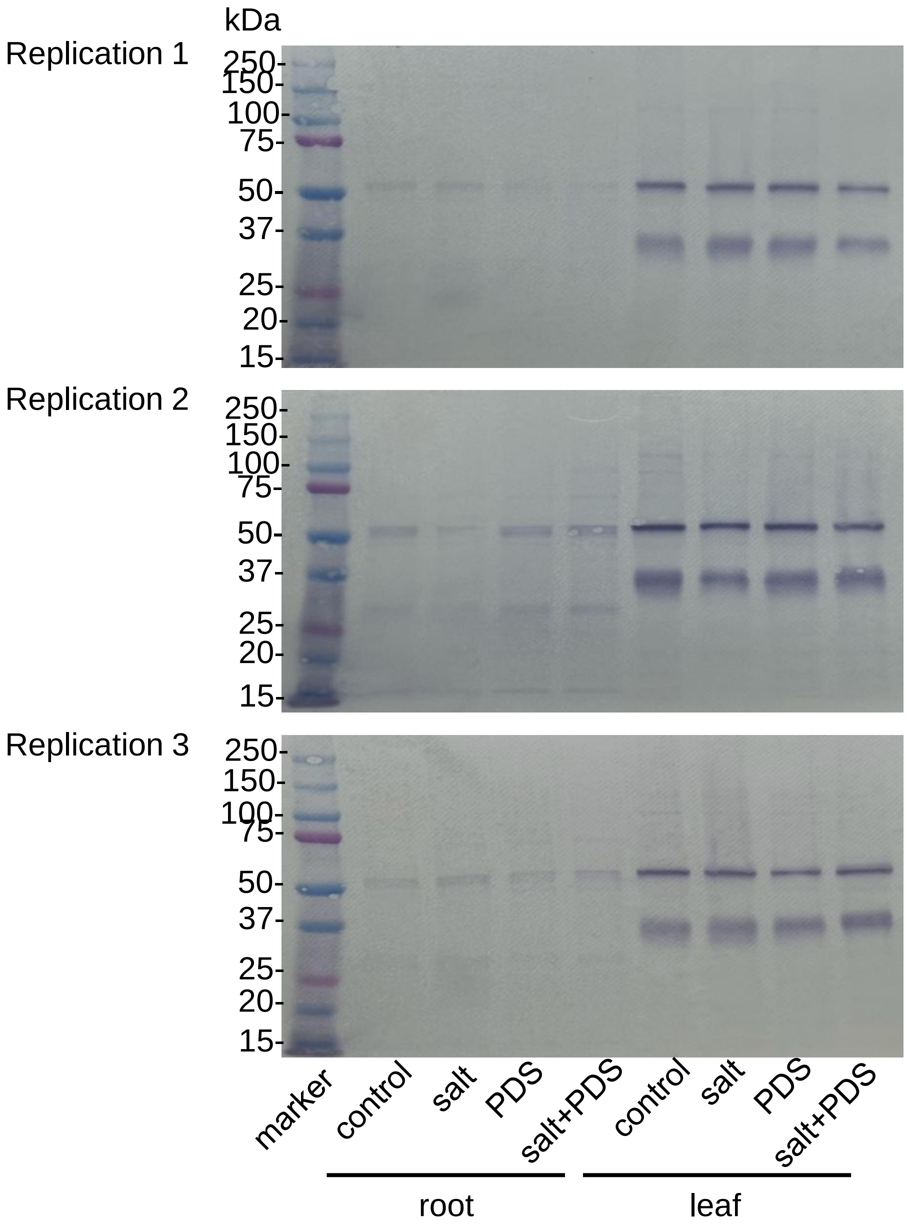

Figure S4. Blots of the entire membrane with anti-GR antibody, which were used in Figure 5B.

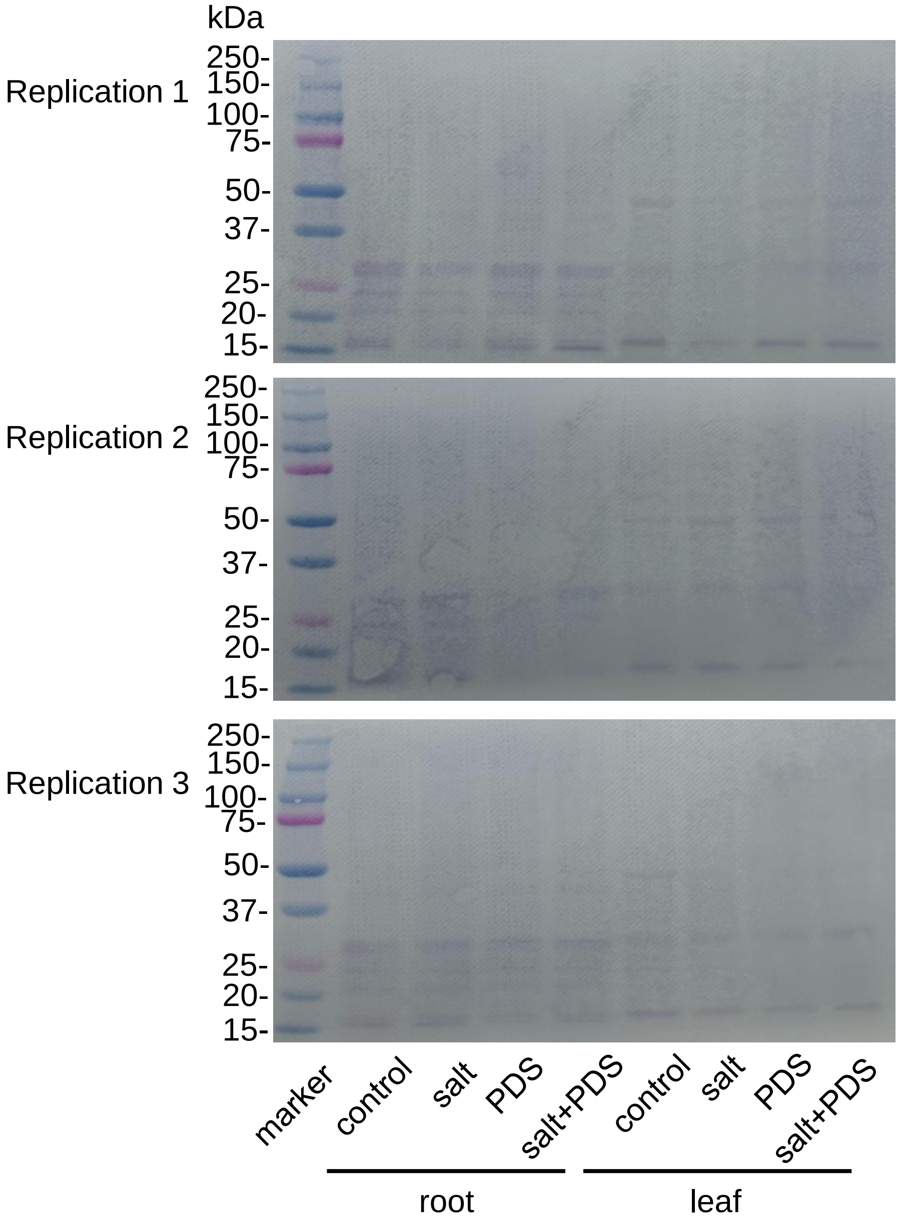

Figure S5. Blots of the entire membrane with anti-SOD antibody, which were used in Figure 5C.

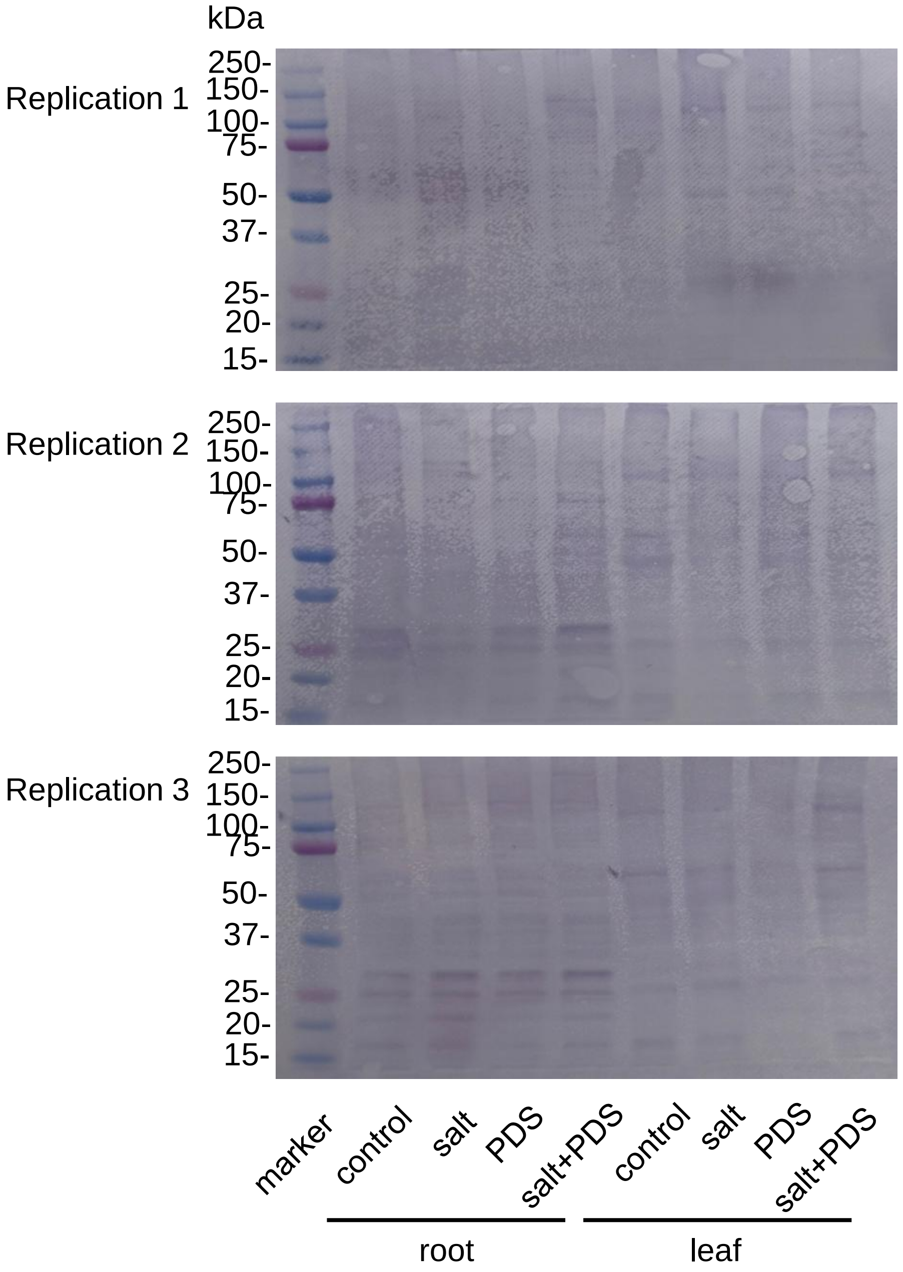

Figure S6. Blots of the entire membrane with anti-PRX antibody, which were used in Figure 5D.

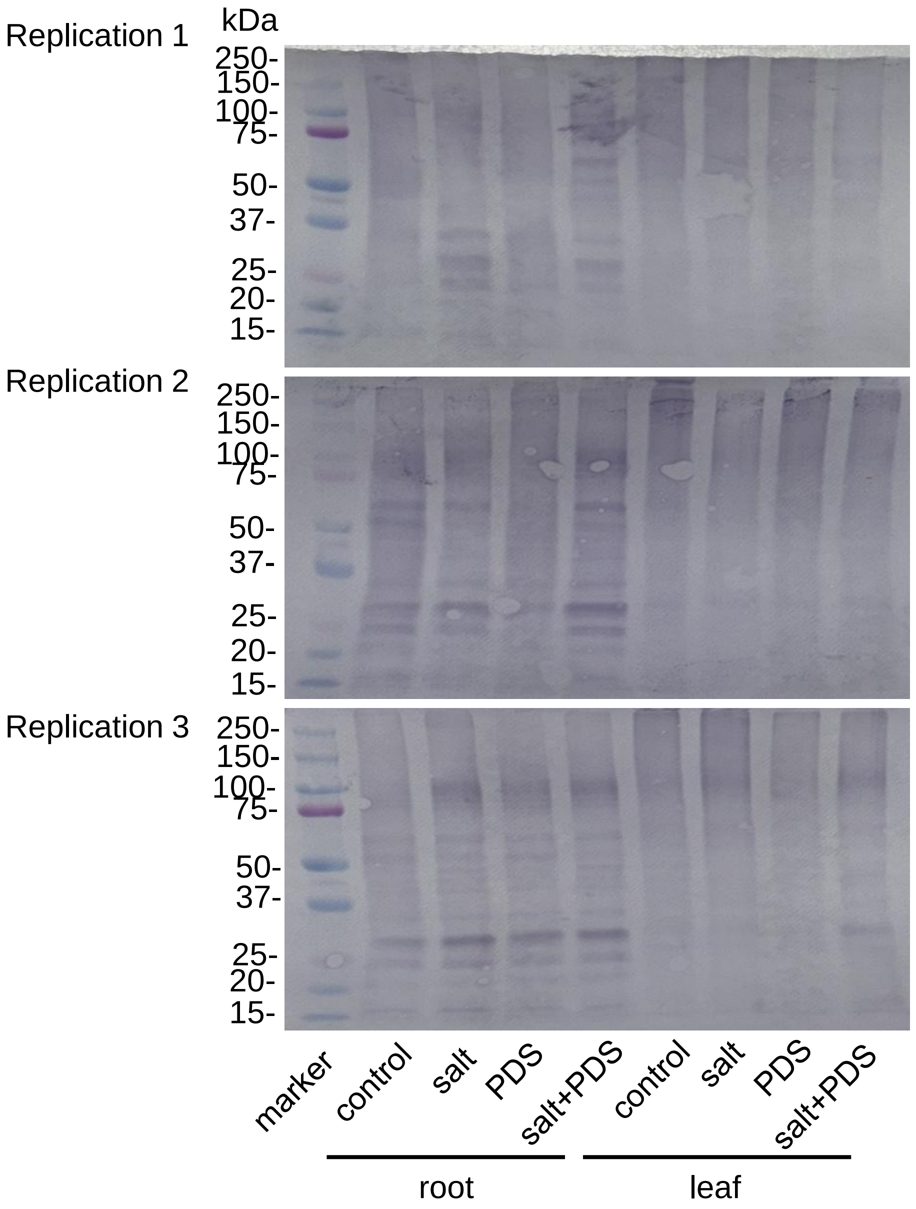

Figure S7. Blots of the entire membrane with anti-H^+-ATPase antibody, which were used in Figure 7A.

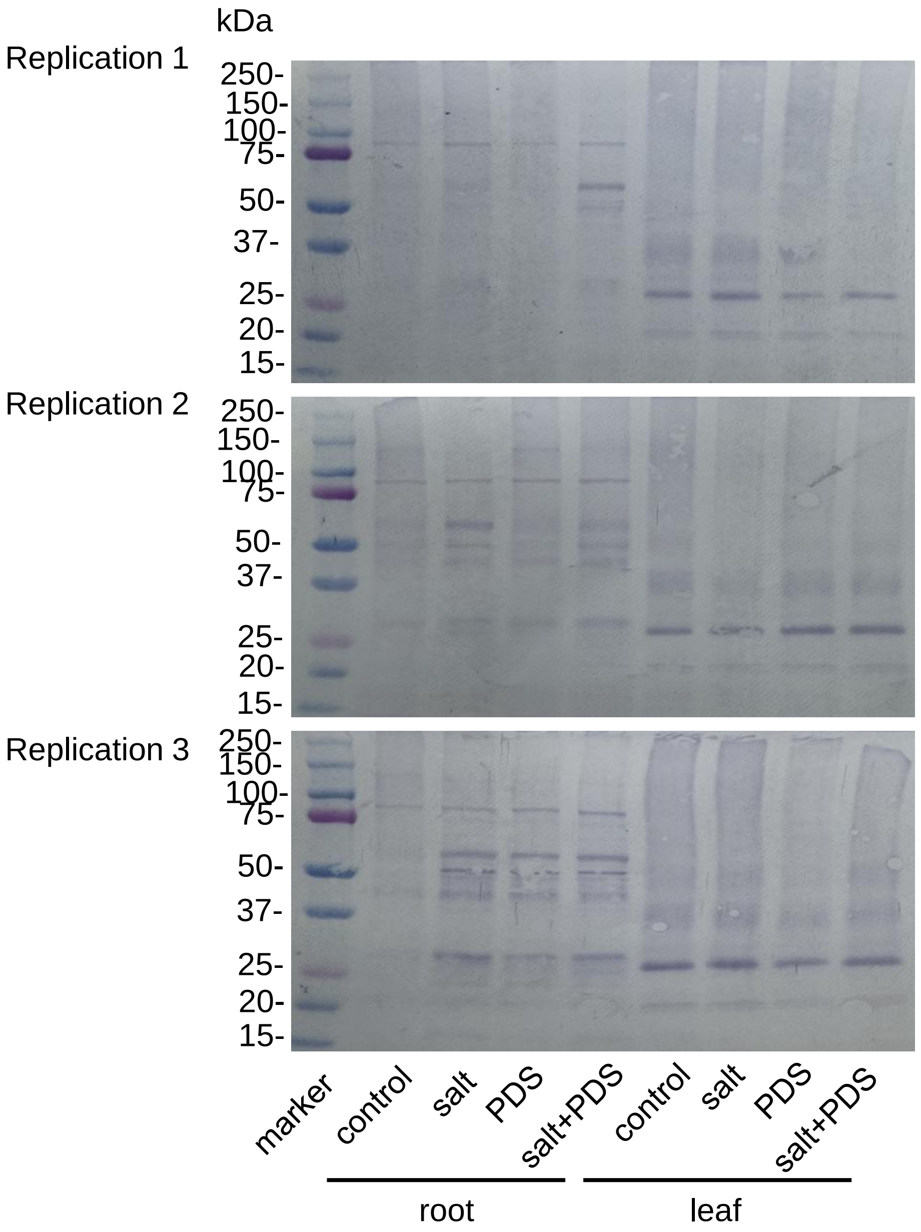

Figure S8. Blots of the entire membrane with anti-aquaporin antibody, which were used in Figure 7B.

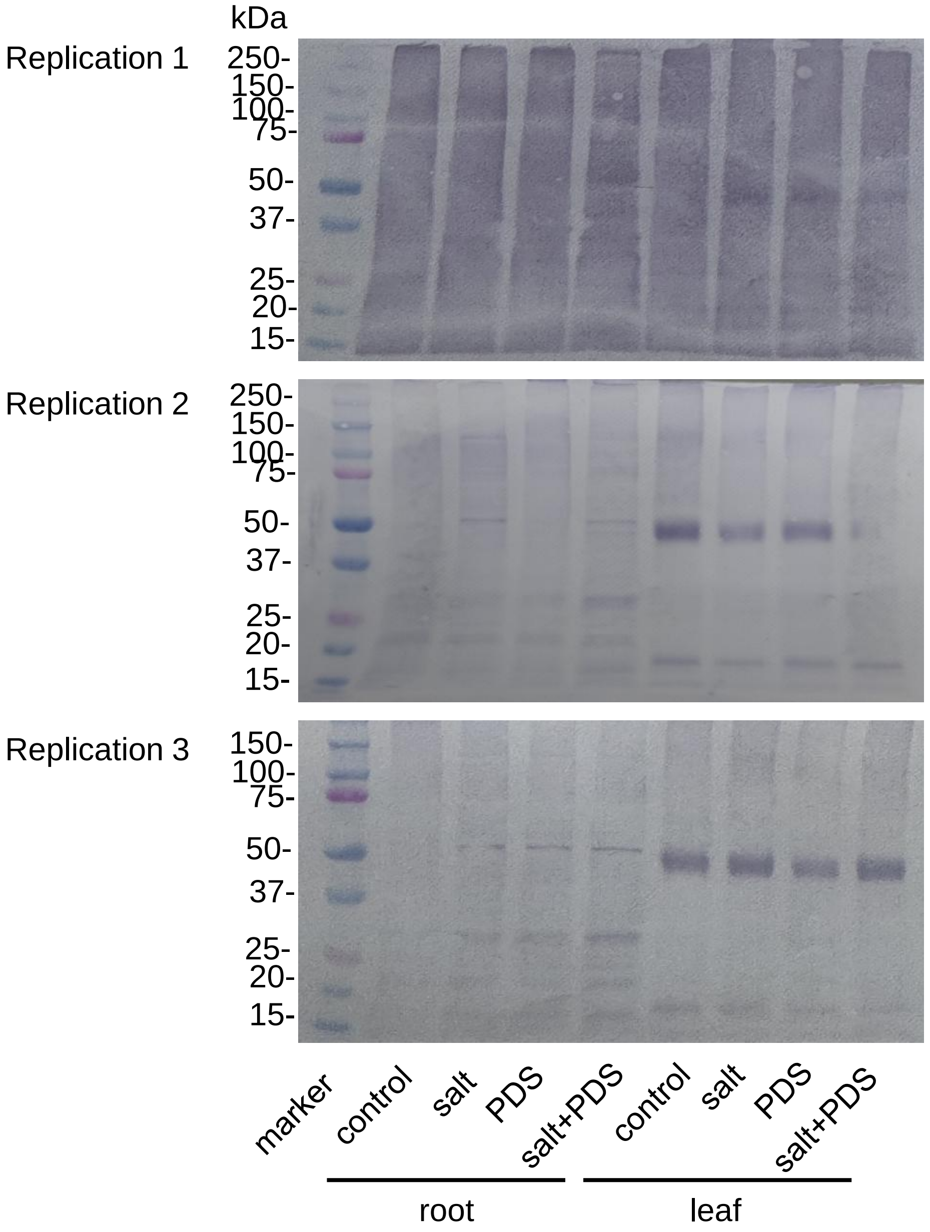

Figure S9. Blots of the entire membrane with anti-V-ATPase antibody, which were used in Figure 7C.

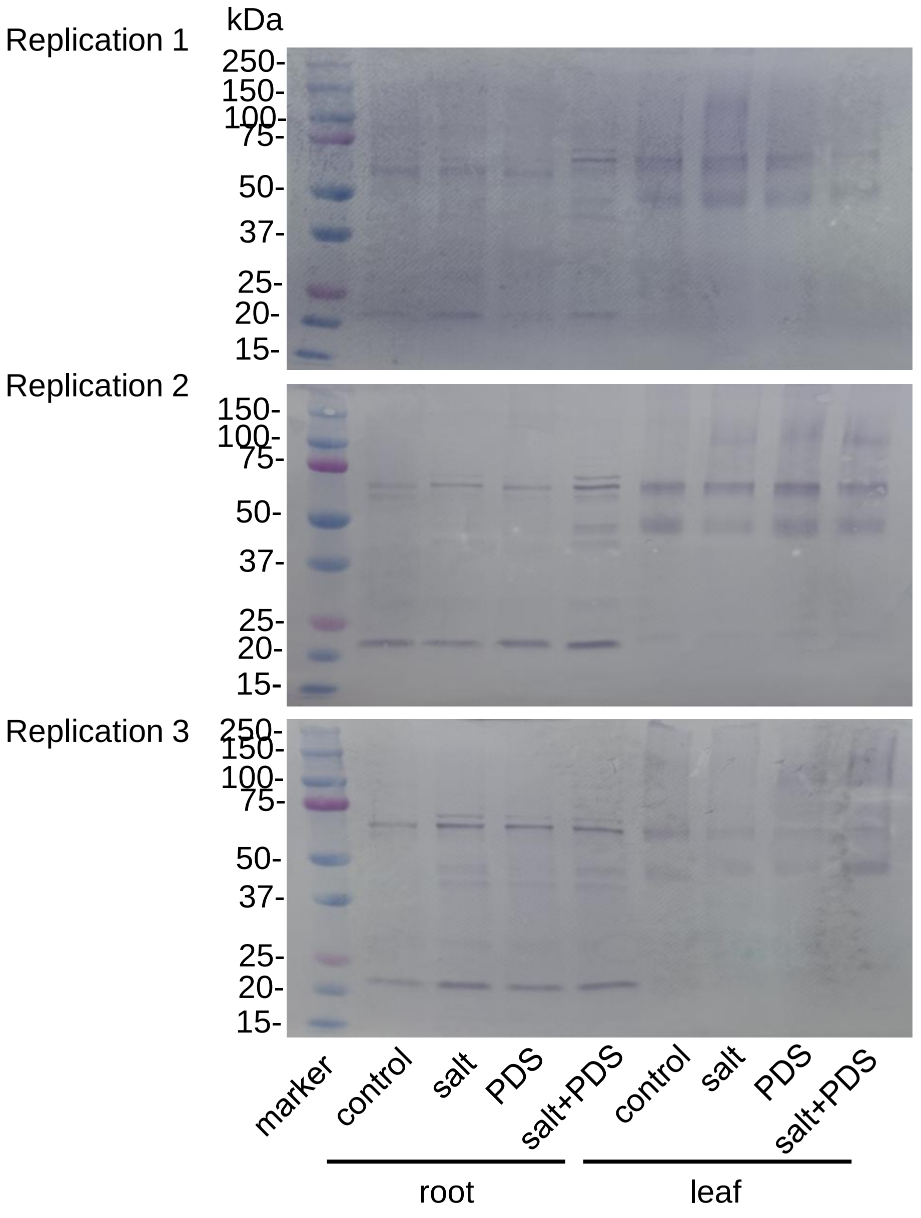

Figure S10. Blots of the entire membrane with anti-calnexin antibody, which were used in Figure 7D.
